# Elucidating the Structure and the Impact of Synthesis Methods on the Flexibility of the Metal‐Organic Framework MIL‐88 A (Fe) During Water Capture

**DOI:** 10.1002/smll.202506833

**Published:** 2025-09-10

**Authors:** Timo Manitz, Fabian Heck, Sri Rezeki, Kristina Gjorgjevikj, Shun Tokuda, Sebastian Bette, Stefano Canossa, Desirée Leistenschneider, Simon Krause

**Affiliations:** ^1^ Nanochemistry Department Max Planck Institute for Solid State Research Heisenbergstraße 1 70569 Stuttgart Germany; ^2^ Department of Chemistry University of Stuttgart Paffenwaldring 55 70569 Stuttgart Germany; ^3^ Department of Chemistry University of Munich (LMU) Butenandstraße 5‐13 81377 Munich Germany; ^4^ Institute for Technical Chemistry and Environmental Chemistry Friedrich‐Schiller University Jena Philosophenweg 7a 07743 Jena Germany; ^5^ Department of Chemistry and Applied Biosciences ETH Zürich Vladimir Prelog Weg 1 Zurich 8093 Switzerland; ^6^ Center for Energy and Environmental Chemistry Jena (CEEC Jena) Philosophenweg 7a 07743 Jena Germany; ^7^ Institut für Anorganische Chemie II Universität Ulm Albert‐Einstein‐Allee 11 89081 Ulm Germany

**Keywords:** flexible metal‐organic framework, in situ diffraction, reproducibility, THz Raman, water adsorption

## Abstract

Flexible metal‐organic frameworks (MOFs) have emerged as a new generation of porous materials and are considered for various applications such as sensing, water or gas capture, and water purification. MIL−88 A (Fe) is one of the earliest and most researched flexible MOFs, but to date, there is a lack in the structural aspects that govern its dynamic behaviour. Here, we report the first crystal structure of DMF‐solvated MIL−88 A and investigate the impact of real structure effects on the dynamic behaviour of MIL−88 A (Fe), particularly upon water adsorption. Four differently synthesized materials are studied with powder X‐ray diffraction (PXRD), THz Raman spectroscopy, and N_2_ sorption. The very high water vapor sorption capacity is probed by utilizing in situ humidity PXRD and calorimetric cycling studies. At least four different crystallographic phases are identified during the sorption measurements and a structural concept and models are developed that explain the changes in the XRD patterns. From this, it is derived that MIL−88 A (Fe) is an excellent material for a new concept of responsive water harvesting technologies.

## Graphical abstract

MIL−88 A is first described in 2004 and consists of a trinuclear iron cluster and fumaric acid as a linker, forming a porous structure. The flexibility of MIL−88 A upon uptake of water, with an expansion in unit cell volume of up to 85%, has been known since 2005. However, no detailed structural models and fundamental understanding of the intermediate structures involved in the swelling process have been proposed. Sorption experiments, in situ powder X‐ray diffraction (PXRD) and Raman spectroscopy are performed under variable humidity conditions to probe the swelling behavior of MIL−88 based on different synthesis conditions. Three new structures are identified that can be assigned to the high water capacity of the framework and, for the first time, correlate this dynamic behavior to the synthesis procedure, which allows to utilize these materials as responsive water harvesting platforms.

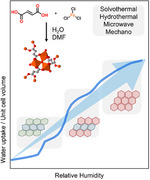

## 1 Introduction

Constructing metal‐organic frameworks (MOFs) via reticular chemistry is supposedly simple: The combination of a metal ion or cluster with an appropriate organic linker forms a 2D or 3D net‐like structure with long‐range order and nanopores. The underlying geometry and connectivity of the respective molecular building blocks dictate the overall framework structure. This approach can fine‐tune structural properties for functional optimization in applications such as atmospheric capture of carbon dioxide^[^ ^1^ ^]^ and water harvesting.^[^ ^2^ ^]^ The majority of known MOFs are considered as rigid, highly ordered frameworks that withstand intermediate hydrostatic pressures and can reversibly adsorb and desorb gases or water vapor. However, real structure effects and intrinsic framework dynamics (flexibility, linker rotation, etc.) strongly impact idealized structure‐property‐function relationships. A few MOFs are even known to exhibit large‐scale structural transformations of the framework lattice as a response to external stimulation or internal adsorption‐induced stress. Common framework deformations in these so‐called flexible MOFs are based on the deformation of the linker (e.g., buckling),^[^ ^3^ ^]^ a change in the geometry of the coordination bond (e.g., hinging of carboxylates),^[^ ^4^ ^]^ or a change in the coordination geometry of the inorganic node itself.^[^ ^5^ ^]^ These adsorption‐induced structural deformations lead to various levels of expansion or contraction of the crystal lattice and pore volume, resulting in gate‐opening, breathing, or swelling transitions.^[^ ^6^ ^]^ These cooperative structural transitions can improve performance in deliverable gas capacity,^[^ ^7^ ^]^ sensing,^[^ ^8^ ^]^ and gas separation^[^ ^9^ ^]^ compared to rigid frameworks and can thus be considered as functional dynamics.^[^ ^10^ ^]^ From a mechanistic perspective, breathing and gate‐opening MOFs are usually bistable systems with energetically highly separated states that can be stabilized by adsorption interactions.^[^ ^11^ ^]^ However, a recent computational analysis of the whole energy landscape during the adsorption process in the breathing of DUT‐49 showed the stabilization of a wide range of different structures with slight variation in unit cell volume in a narrow gas loading regime.^[^ ^12^ ^]^ Such behavior is more reminiscent of swelling transitions with gradual expansion or contraction of the unit cell volume under preservation of long‐range order, reflecting a shallow energy landscape with many local minima.^[^ ^13^ ^]^ The resulting enhanced structural space renders swelling MOFs more sensitive and adaptive toward small changes in adsorption conditions compared to breathing or gate‐opening MOFs. Utilization of this aspect toward enhancing the performance in applications such as water harvesting, actuation,^[^ ^14^ ^]^ and sensing in different ranges of relative humidity (RH), however, remains scarcely explored.

The first reported swelling MOF is MIL−88 A (Fe), which was obtained as a microcrystalline powder of hexagonal rod‐like crystals with a framework of **acs** topology based on hexaconnected [M_3_O(CO_2_)_6_X](M = Fe; X = acetate or Cl^−^) clusters bridged by fumaric acid (FA).^[^ ^15^ ^]^ Both building blocks are abundant in natural sources, fulfilling one important criterion for large‐scale production of MIL−88 A.^[^ ^16^ ^]^ Despite an established structure from Serre et al.^[^ ^15^ ^]^ on powder, no single crystal analysis of MIL−88 A (Fe) has been reported to the best of our knowledge. Mellot‐Draznieks et al. investigated the swelling behavior of desolvated MIL−88 A in the presence of water and other liquids.^[^ ^17^ ^]^ They reported an increase in the unit cell volume by 85% from the dried, contracted pore (*cp*) form to the fully solvated open pore (*op*) form, depending on the size and polarity of the guest molecules. A particularly pronounced lattice expansion was observed upon the uptake of water compared to aliphatic alcohols. This renders MIL−88 A (Fe) an interesting candidate for water purification,^[^ ^18^ ^]^ sensing,^[^ ^19^ ^]^ and water‐responsive actuation.^[^ ^20^ ^]^ Serre et al. established the isoreticular MIL−88 series (MIL−88 B, ‐C, and ‐D) with elongated linker backbones. All materials in this series exhibit swelling behavior.^[^ ^4^ ^]^ Recent theoretical studies of the energy landscape of MIL−88 B provide a detailed analysis of thermodynamic and kinetic aspects that govern the uncommon gradual structural transitions. A very shallow energy landscape with many local minima upon adsorption of methanol explains the ability of MIL−88 B to adapt its structure upon guest uptake by stepwise pore expansion.^[^ ^21^ ^]^ However, these simulations are based on idealized structures within periodic boundary conditions and have to date not been correlated with experimentally determined structural models. Over the past 10 years, real structure effects such as vacancy defects, particle size, and morphology were found to strongly impact the structural response of flexible MOFs,^[^ ^22^ ^]^ both in experiment^[^ ^23^ ^]^ and theory.^[^ ^24^ ^]^ It is expected that such real structure effects are particularly pronounced for swelling MOFs given their shallow energy landscape, yet, to date, no such analysis has been performed neither theoretically nor experimentally on MIL−88 A and related materials. This is particularly interesting given the extensive applications and various synthesis procedures that are reported for MIL−88 A: The first synthesis reported by Serre et al.^[^ ^15^ ^]^ was carried out solvothermally, where basic iron acetate, produced by the route of Dziobkowski et al.,^[^ ^25^ ^]^ and fumaric acid were reacted in a mixture of water and methanol at 373 K. Chalati et al. varied the reaction method, temperature, time, pH, and concentration to tailor the particle size of MIL−88 A (Fe) in the range of hundreds of nanometers up to a few micrometers.^[^ ^26^ ^]^ Considering the early discovery of MIL−88 A and the extensive existing literature on the material, comprising over 310 publications to date, in‐depth research on the underlying structure and guest‐responsive transformations is nonexistent. In addition, the influence of real structure factors such as particle size and morphology on the structural dynamics, especially as a function of water adsorption, has not been investigated to date.

In this contribution, we analyze for the first time the impact of real structure effects due to different synthesis methods on the dynamic behavior of MIL−88 A (Fe), particularly upon water adsorption. We first synthesized and analyzed crystals large enough for single‐crystal X‐ray diffraction (SCXRD) and present the first single‐crystal structure of DMF‐solvated MIL−88 A. We further synthesized bulk powders of MIL−88 A by solvothermal, hydrothermal, mechanochemical, and microwave‐assisted methods. We characterized all samples by powder X‐ray diffraction (PXRD), N_2_ adsorption at 77 K, thermogravimetric analysis with mass spectrometry (TGA/MS), scanning electron microscopy (SEM), Raman spectroscopy and energy dispersive X‐ray spectroscopy (EDX) to show the influence of the reaction method on material properties such as crystallinity, crystal size and morphology, and composition. We furthermore probed the swelling behavior upon water adsorption by in situ humidity PXRD, Raman spectroscopy, and water vapor sorption measurements. We demonstrate that all MIL−88 A (Fe) samples show multistep‐like water adsorption isotherms and wide hysteresis due to a combination of gradual and stepwise structural transformations. We investigated the adsorption‐desorption cycle stability over 100 cycles by calorimetric adsorption methods. Additionally, we propose two new structural models for the intermediate phases of MIL−88 A observed during the swelling process, derived from in situ PXRD. Our results show that the structural dynamics enhance the water adsorption capacity in critical humidity ranges, paired with high cycle stability and hysteretic behavior that might facilitate efficient water release. However, we also show that these aspects strongly depend on real structure effects linked to the synthesis method. As such, we provide for the first time a unique insight into the structural response of the swelling MOF MIL−88 A toward water adsorption, but highlight that these features strongly depend on the materials preparation and synthesis conditions due to real structure effects.

## 2 Results and Discussion

### 2.1 Single Crystal Structure

**Figure 1:**
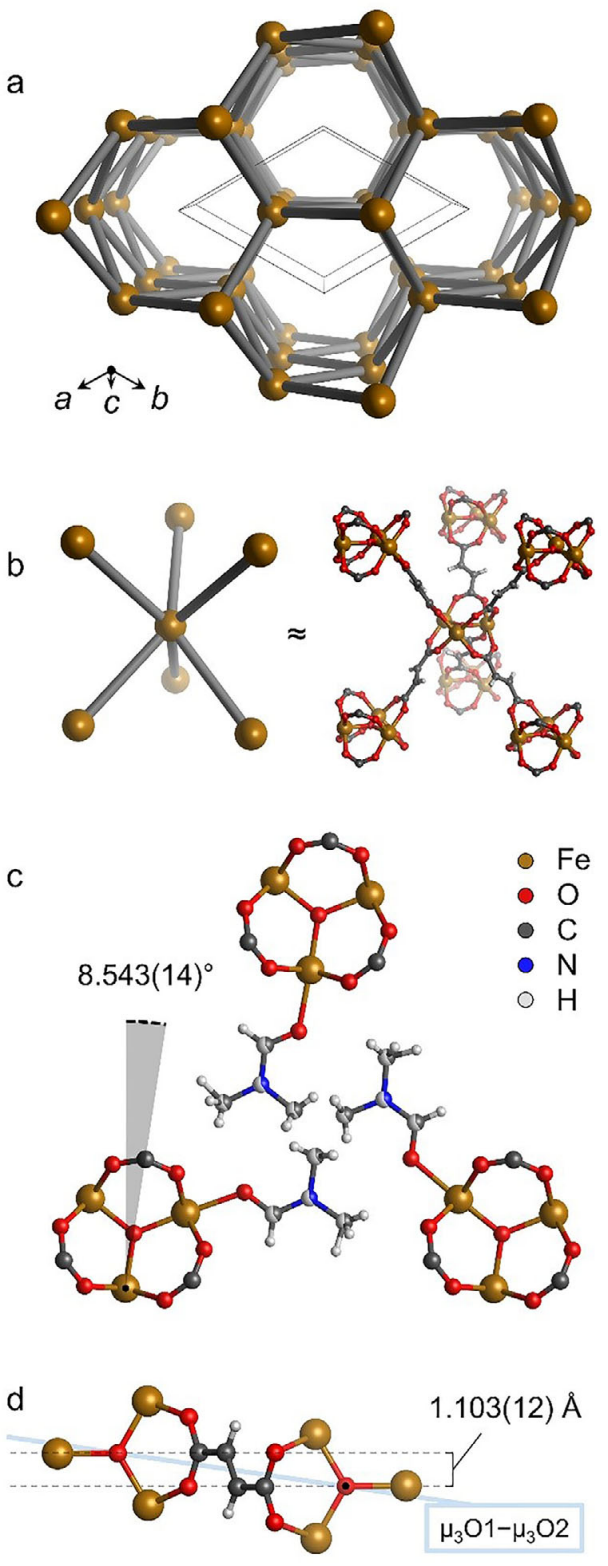
Salient features of MIL−88 A structure. A topological view (a; unit cell in solid line) shows the trigonal prismatic geometry of node connections (b). The SBUs plane highlights a peculiar SBU tilt (c) correlating with the non‐collinearity of linker carboxylates (d).

The growth of sufficiently large single crystals of MIL−88 A (Fe) with a bipyramidal rod‐like morphology was achieved by solvothermal synthesis in *N,N*‐dimethylformamide (DMF). They allowed for structural analysis by synchrotron single crystal X‐ray diffraction at 100 K. Our refined structure (space group: *P*6_3_/*m*; **Figure** **1**) shows significant differences compared to the previously published structural model based on PXRD data at room temperature.^[^ ^15^ ^]^ Most notably, while the previously reported structure contains fumarate linkers with a carbon backbone in a plane orthogonal to those of the carboxylate groups, in our model, fumarate species are completely planar (dihedral angle 0.0(7)°), which suggests an effective delocalization of electron density across the linker. Second, the (COO)_6_Fe_3_O nodes are not perfectly aligned along the [1‐10] direction (long diagonal of the unit cell) but are instead tilted by 8.543(14)° (Figure 1c). This tilt is also present in the original structure (space group *P*–62*c*), but amounts to a much smaller value 0.9(7)°, while in the other crystal structure reported for scandium MIL−88 A (determined from single crystal data at 150 K)^[^ ^27^ ^]^ the angle is 8.21(2)°, thus, closely approaching our Fe‐based model. While temperature conditions can influence both the planarity of linkers and the SBU tilting, these characteristics were also reported for the room temperature structure model of the fumarate‐based MOF‐801.^[^ ^28^ ^]^ Here, the orientation of octahedral Zr SBUs^[^ ^29^ ^]^ deviates from their more symmetrical arrangement in the terephthalate‐based parent MOF known as UiO‐66. The observations described above suggest that the lack of co‐linearity of the two C─C bonds in the C‐CO_2_ groups of fumarate linkers plays a dominant role in the emergence of SBU tilting in MIL−88 A and its underlying flexibility. Such influence might apply to many other MOFs that have not been structurally characterized yet, or whose structural models have been subjected to excessive symmetry restraints.

During the publication of this work, Schumann et al.^[^ ^30^ ^]^ reported a strategy to produce and analyze single crystals of MIL−88 A by a different synthetic approach. Their reported space group is consistent with ours, and the unit cell volume (2046 Å^3^) exhibits a comparable value to the unit cells we have determined through Pawley refinement off hydrated MIL−88 A samples (see **Table** **1**) and can therefore also be assigned to the open pore phase. The unit cell we report with our single crystal structure is slightly smaller (1910 Å^3^). This shows the flexibility of MIL−88 A.

**Table 1:** Synthesis conditions of MIL−88 A and the respective crystallographic analysis and crystal size distribution. Unit cell volumes determined by Pawley refinement from PXRD patterns, mean crystal dimensions determined by analysis of SEM images.

Synthesis method	Solvothermal	Hydrothermal	Microwave‐assisted	Mechanochemical
Sample code	**MIL−88 A Sol**	**MIL−88 A Hyd**	**MIL−88 A Mic**	**MIL−88 A Mec**
Reaction time (h)	24	24	1	0.16 (10 min)
Reaction temperature (K)	373	373	373	298
Solvent synthesis	DMF	Water	DMF	Solvent‐free
Unit cell volume as made (Å^3^)	1947	2094	1940	2016
Unit cell volume water (Å^3^)	2102	2102	2100	2100
Mean crystal length (µm)	3.87 ± 1.53	4.20 ± 3.66	1.99 ± 0.93	non quantifiable
Mean crystal width (µm)	3.37 ± 1.26	1.34 ± 0.65	1.01 ± 0.51	non quantifiable

### 2.2 Synthesis Method Variation

We synthesized MIL−88 A as bulk powders under solvothermal (DMF as solvent; denoted as **MIL−88 A Sol**) and hydrothermal (denoted as **MIL−88 A Hyd**) reaction conditions as reported by Chalati et al.^[^ ^26^ ^]^ Additionally, we replicated the microwave‐assisted solvothermal reaction in DMF as previously described by Chalati et al.^[^ ^26^ ^]^ with minor adaptations (for more details, see Supporting Information; denoted as **MIL−88 A Mic**). We also synthesized MIL−88 A using a solvent‐free mechanochemical method (denoted as **MIL−88 A Mec**), as reported by Jeong and Lee.^[^ ^31^ ^]^ These reaction conditions were chosen to investigate a broad range of typical synthesis scenarios, including reaction approaches that address green chemistry by avoiding or saving toxic solvents, high energy consumption, and long reaction times.^[^ ^32^ ^]^ The synthesis conditions and the corresponding sample names are summarized in **Table** **1**.

**Figure 2:**
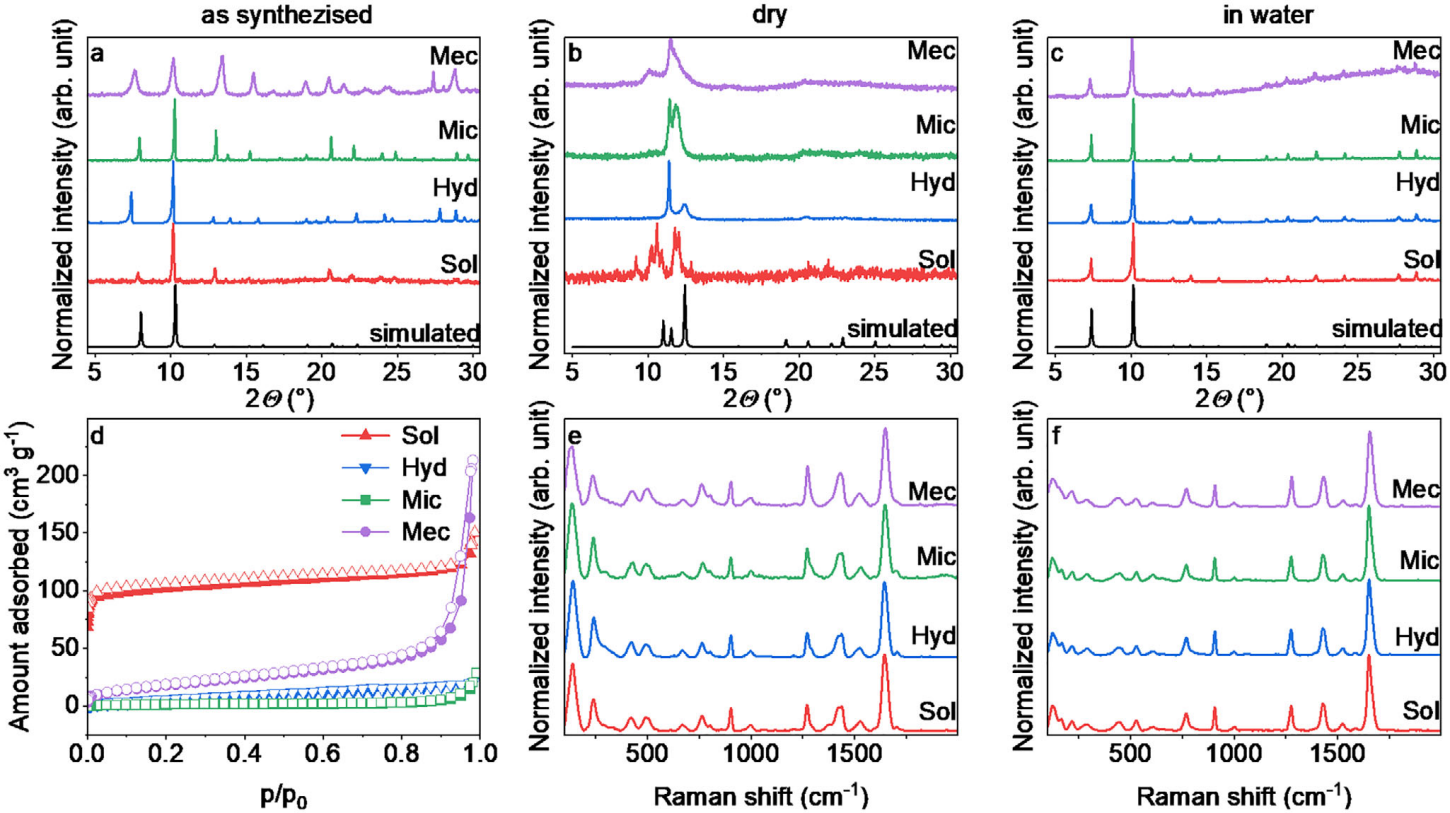
PXRD patterns of as‐synthesized (a), dryed (b), and redispersed in water (c) MIL−88 A synthesized via mechanochemical (purple; MIL−88 A Mec), microwave (green; MIL−88 A Mic) hydrothermal (blue; MIL−88 A Hyd), and solvothermal (red; MIL−88 A Sol) synthesis approach; simulated pattern (black) based on the presented single crystal structure of MIL−88 A and in 2 b with adjusted unit cell parameters according to values from literature.^[^ ^4^ ^]^ d) N_2_ sorption isotherms (77 K), closed symbols: desorption; open symbols adsorption, Raman spectra of the synthesized MIL−88 A after drying (e) and in water (f).

The collected PXRD patterns (**Figure** **2**a) of the four as‐synthesized (as) (DMF or water‐containing) bulk powders indicate the formation of phase pure MIL−88 A (Fe) as demonstrated by Pawley refinement (Figures S3–S6, Supporting Information).^[^ ^15^, ^17^ ^]^

The PXRD pattern of **MIL−88 A Mec as** shows a variation of relative reflection intensities and also peak broadening with a low signal‐to‐noise ratio. The intensity change can be explained by the variation of diffuse electron density in the pores. This phenomenon can be attributed to the utilization of different synthesis solvents or humidity. The broadening indicates formation of small crystal domains reflected by the small average particle size (<200 nm) investigated by SEM. Additionally, NaCl was formed as a by‐product during this synthesis. During the washing of the sample, the NaCl was successfully removed as the reflections of NaCl are absent in Figure S11 (Supporting Information). The PXRD patterns of **MIL−88 A Mec as** and **Mic as** differ in intensity from the simulated pattern. This is due to structural disorder.

Additionally, PXRD patterns of all synthesis approaches show slight variations in the peak positions (Figure 2a), indicating variations in the unit cell dimensions. Pawley fitting of the patterns indicates that the unit cell volumes of the as MIL−88 A samples range from 1940 to 2094 Å^3^. The smallest unit cell volume is observed in **MIL−88 A Mic** and **Sol** (both synthesized in DMF) with 1940 and 1947 Å^3^, respectively, while for **MIL−88 A Mec** the unit cell volume is 2016 Å^3^, and for **MIL−88 A Hyd** 2094 Å3. This difference in unit cell volume can be explained by the solvents used in the reaction, with DMF‐based synthesis resulting in a more contracted structure compared to water‐filled pores with an expanded pore structure.^[^ ^17^ ^]^ We expect the water in the **MIL−88 A Mec** sample to originate from the FeCl_3_ hexahydrate or air moisture.

Upon drying of the samples at 373 K in dynamic vacuum (p < 10^−4^ kPa) for at least 12 h, the PXRD patterns of all samples show drastic changes in peak position, number, and intensity, indicating a large‐scale structural transformation upon guest removal (Figure 2b).

Redispersion of the dry samples in water results in the recovery of the open pore form as demonstrated by the recovered PXRD patterns, showing that MIL−88 A is flexible and does not lose structural integrity during the guest molecule removal (Figure 2c). Especially **MIL−88 A Mec** results in the open pore form with improved reflection sharpness compared to the as form. Pawley fitting indicates that the unit cell volumes of all samples range from 2100 to 2102 Å^3^, similar to the **as MIL−88 A Hyd**.

The N_2_ sorption isotherms are shown in Figure 2d. The isotherms of **MIL−88 A Hyd**, **Mic,** and **Mec** show non‐porous behavior, and **MIL−88 A Mec** shows interparticle adsorption from a partial pressure of 0.8 ongoing. **MIL−88 A Sol** shows a type I(b) behavior. It is known from the literature that the N_2_ uptake varies due to sample synthesis.^[^ ^33^ ^]^

We probed the dried samples with TGA to investigate the thermal stability as well as the content and nature of residual guest species in the pores. The measurements (Figure S17, Supporting Information) show mass loss steps at 373 K, 423 K, 573 K, and between 673 and 723 K. These losses can be attributed to the removal of uncoordinated and coordinated solvent molecules, unreacted linker molecules, and the final degradation of the MOF.^[^ ^34^ ^]^ Furthermore, the **MIL−88 A Sol** and **Mic** samples show in the MS spectra at 440 and 575 K the release of DMF, which was not removed from the structure during the drying procedure. These results show that green chemistry and non‐toxic synthesis solvents are important aspects to consider, since MIL−88 A could be a platform for medical applications as e.g. drug delivery or imaging.^[^ ^35^ ^]^

We further investigate the structural response of MIL−88 A to water exposure by THz Raman spectroscopy. The analysis of both dry and water‐dispersed samples synthesized by different methods (Figure 2e,f, respectively) indicates that the Raman features are not dependent on the preparation procedure. A summary of all peak positions in the Raman spectra of both dry and water‐dispersed samples, as well as their assigned vibrational modes, can be seen in Table S3 (Supporting Information).^[^ ^36^ ^]^

Similar spectral changes were identified for all samples upon pore opening. Most of the low‐frequency region bands, which are mainly attributed to lattice vibrational modes, show a red shift upon dispersion in water. This is to be expected upon pore opening, due to the effect of tensile stress on the structure.

In contrast, bands in the fingerprint region assigned to the inner part of the linker molecule are blue‐shifted, suggesting a structure compression in these regions. No significant changes are observed for the C═O modes.

In addition, we see some band broadening and the appearance of new weak bands, which can be attributed to the influence of water on the electronic cloud of the building units.

These changes are more clearly illustrated in Figures S30–S33 (Supporting Information), which compare the dry samples with the corresponding sample dispersed in water.

The distinct differences in the Raman spectra of the open and closed pore structures of MIL−88 A underscore the effectiveness of Raman spectroscopy as a rapid and non‐destructive method for distinguishing MOF polymorphs.

It is important to note that the Raman analysis of one dry mechanochemically synthesized sample revealed the presence of multiple phases in small amounts within the capillary. The spectra of these impurities are shown in Figure S34 (Supporting Information). Identifying and assigning the vibrational modes of these trace phases is beyond the scope of this work.

### 2.3 Humidity Response

**Figure 3:**
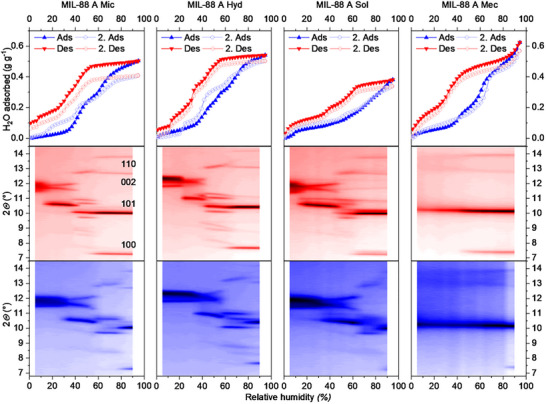
Water vapor sorption isotherms (upper row; color code: red desorption and blue adsorption) and *in* *situ* XRD pattern of MIL−88 A during desorption (middle row; color code: low intensity white, middle intensity red, and high intensity black, gradual increase) and adsorption (bottom row; color code: low intensity white, middle intensity blue, and high intensity black, gradual increase).

We performed water vapor sorption experiments at 298 K to investigate the difference in water capacity of MIL−88 A samples at different levels of humidity. All sorption isotherms (**Figure** **3**, top row) show a multi‐step behavior during adsorption and desorption of water, but the overall uptake, isotherm shape, and hysteresis vary among all samples. While **MIL−88 A Hyd** and **Mic** exhibit a similar isotherm shape, with **Hyd** showing a slightly higher uptake, **MIL−88 A Sol** and **Mec** are different, but show similar features to each other. **MIL−88 A Hyd** and **Mic** start with a moderate uptake in the first cycle and show two increased water uptake steps starting from 30% RH. In the second cycle, an additional uptake step can be observed at around 20% RH. During the desorption, both samples show increased release of water from 55% RH with steps at 35–20% RH and 10% RH. Changes between the first and the second cycle are not observed. In comparison to that, the samples **MIL−88 A Sol** and **Mec** already show an increased water uptake at low relative humidity (<10% RH). **MIL−88 A Sol** shows a continuous increase of uptake during the first cycle and additional steps at 45 and 65% RH in the second cycle. **MIL−88 A Mec** shows two steps in the first cycle (35 and 50% RH), which are not as prominent in the second cycle. Additionally, inter‐particle condensation is observed at high RH (>80%) due to the smaller particle size in **Mec**. The overall water uptake varies for each sample (0.38 g g^−1^ for **MIL−88 A Sol**; 0.50 g g^−1^ for **MIL−88 A Mic**; 0.62 g g^−1^ for **MIL−88 A Mec**; 0.54 g g^−1^ for **MIL−88 A Hyd**) and is in line with the best performing reported MOFs for ambient air water harvesting.^[^ ^37^ ^]^ Although most water harvesting applications should be designed for arid climates (17–32% RH),^[^ ^37^ ^]^ it is important to note that the highest currently reported water capture capacity of a MOF (Cr−soc−MOF−1) is 1.95 g g^−1^ in the range between 60 and 75% RH.^[^ ^38^ ^]^ In MIL−88 A, steps and a wide hysteresis are also present only at higher relative humidity levels (40–80% RH), which could be used for water harvesting applications in humid climate regions. In fact, to date, no technology utilizes framework dynamics as a functional^[^ ^39^ ^]^ property to alter the water harvesting performance, similar to proposals on gas separation and storage.^[^ ^16^ ^]^

The different water isotherms and the measured overall water uptake demonstrate that the performance of the water harvesting ability strongly depends on the synthesis procedure employed. To identify the structural origin of the stepped isotherm and hysteresis and their dependence on the synthesis method, we performed in situ PXRD under variable RH at 298 K in continuous flow (Figure 3, middle and bottom row), starting from water‐filled samples down to low RH and subsequent RH increase. In all four samples, the XRD patterns at high relative humidity (90%) values indicate the open pore form of MIL−88 A, matching the PXRD patterns of water‐suspended MIL−88 A (Figure 2c). At low relative humidity values (5%), the diffraction patterns reflect the dry form. In the RH‐regime in between, we observe several well‐defined stepped structural transformations with gradual lattice changes within the steps.

While **MIL−88 A Mic**, **Hyd**, and **Sol** are showing similar behavior during the desorption and adsorption process, **MIL−88 A Mec** differs from the three other samples. **MIL−88 A Mec** shows a decrease in reflection intensity until the peaks are partially or completely absent, and the 101 diffraction line becomes smaller in intensity without changing its diffraction angles. This indicates that **Mec** is locked in the open phase, and the desorption heat is insufficient to trigger a phase transition. Similar size‐dependent flexibility has already been shown for DUT‐8 in the nanometer regime.^[^ ^40^ ^]^ In contrast, the positions of the individual peaks in the other samples undergo substantial changes, with the emergence and disappearance of new reflections during the course of the experiment. For all samples, the transitions during desorption occur at a lower relative humidity compared to that during adsorption, matching the hysteresis in the vapor isotherms.

Although **MIL−88 A Mic**, **Hyd**, and **Sol** demonstrate comparable behavior during the desorption and adsorption process, there are observable discrepancies between the samples produced with DMF (**MIL−88 A Mic**, **Sol**) and the sample produced in water (**MIL−88 A Hyd**).

Selected XRDs from the in situ XRD measurements are presented in the Figures S18–S21 (Supporting Information), showing the different XRD patterns during the adsorption (10, 30, 55, and 85% RH) and desorption process (85, 70, 50, and 20% RH).

**Figure 4:**
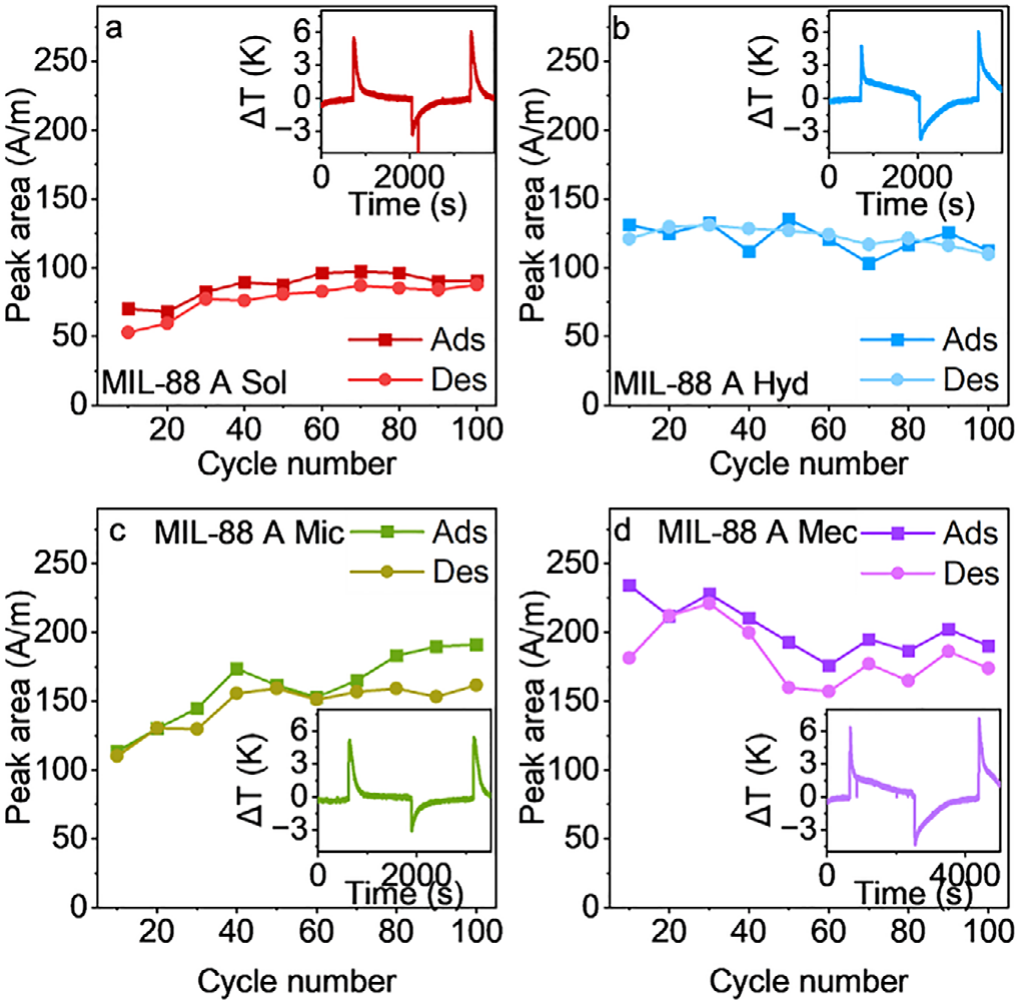
Compound stability test using Infrasorp for 100 cycles at 90% RH for (a) MIL 88 A‐Sol, b) MIL 88 A‐Hyd, c) MIL 88 A‐Mic, and (d) MIL 88 A Mec. The insets show the first adsorption and desorption cycle.

The combination of water sorption and in situ XRD data shows the extreme swelling of MIL−88 A (Fe) toward different humidity levels in a single adsorption‐desorption experiment. Of the four samples, **Mil‐88 A Mic** shows the highest decrease in adsorption capacity and a change in the isotherm shape in a second adsorption cycle (**Figure** 3). The reduced uptake can be explained by the fact that the sample underwent neither outgassing between the two cycles nor a complete removal of water molecules from the samples by vacuum. For the altered isotherm shape, it is hypothesized that the sample undergoes activation, a process that occurs during the initial adsorption cycle. To further investigate the stability of the materials upon extended cycling, we used an optical calorimeter (INFRAsorp) to record the temperature increase in the measurement cell due to heat formation during 100 dynamic water adsorption‐desorption cycles (**Figure** **4**). This approach has been proven to be a powerful tool to rapidly test the stability of MOF materials during water adsorption and desorption^[^ ^41^ ^]^ as well as to determine the cycling stability of flexible MOFs.^[^ ^42^ ^]^ In principle, heat formation during adsorption mainly relates to two factors: the number of water molecules adsorbed and the strength of interaction between the adsorptive and the adsorbate.^[^ ^43^ ^]^ Due to heat dissipation and cooling in the dynamic measurement factors, such as the particle size and bed packing, but also structural transformations, can influence the temperature increase that is recorded by the calorimeter.^[^ ^42^, ^44^ ^]^

In the first water adsorption cycle, **MIL−88 A Mec** demonstrates the highest heat generation during the first adsorption process, which is equivalent to the peak area (*A/m*) of 207 K s mg^−1^ (Figure 4d). Also, this indicates that **Mec** is locked in the open state, and the phase transition does not occur. **MIL−88 A Hyd** exhibits a peak area of 144 K s mg^−1^ (Figure 4b), followed by **MIL−88 A Sol** (Figure 4a) with an area of 114 K s mg^−1^, and **MIL−88 A Mic** at 67 K s mg^−1^ (Figure 4c) reflecting the total uptakes determined in the water adsorption experiments (Figure 3) and indicating the comparability of the sample series. (Figure S12, Supporting Information).

Upon cycling, **MIL−88 A Sol** (Figure 4a) demonstrated a slight increase in peak area across the cycles (although with the lowest overall capacity). In addition, **MIL−88 A Mic** (Figure 4c) showed a more apparent increasing peak but was still lower than **MIL−88 A Mec** at the 100^th^ peak. Moreover, the **MIL−88 A Mec** showed the most substantial heat formation during water adsorption among the tested materials. We assume that this heat is not used for a structural transformation, as in the other samples, and that the **MIL−88 A Mec** is locked in the open phase, as the heat is not sufficient to trigger a phase transition. It is noteworthy that the peak area is strongly influenced by the equilibration time of the adsorption and desorption cycle. In case of not fully equilibrated processes, peak areas are reduced due to an incomplete adsorption/desorption process. Additionally, we noticed changes in the slope of the temperature decays. The minor differences observed in the data from various experiments suggest that the synthesis methods impact the material's performance significantly. The deviations throughout the cycling of a single sample are mostly within the margin of error of the applied method.

Static volumetric water adsorption (Figure 3) revealed step‐wise adsorption of water due to structural changes in the material as demonstrated by in situ PXRD in continuous flow of water vapor. To elaborate on the effect of the structural transitions on the Infrasorp temperature profiles, we tested adsorption at different humidity levels of 20, 30, 55, 70, and 90% (Figures S13–S16, Supporting Information). Temperature profiles at lower RH (20–55%) show a regular thermal decay comparable to water uptake in microporous systems^[^ ^45^ ^]^ with purely physisorption‐like interactions. However, at higher RH (70–90%), we observed the pronounced evolution of a stepped heat profile, indicating the stepwise adsorption process likely due to the structural pore expansion at higher water uptake. This trend is present for all samples investigated, with the pronounced differences in total water uptake discussed before. It needs to be noted that this profile is the convolution of the highly non‐linear exothermal adsorption process, combined with the endothermal process of structural expansion of the framework, which itself is strongly dependent on the water uptake. Our analysis indicates that this complex mechanism might provide access to strongly non‐linear adsorption chiller processes^[^ ^46^ ^]^ for which a strong response in a specific RH range is required.

**Figure 5:**
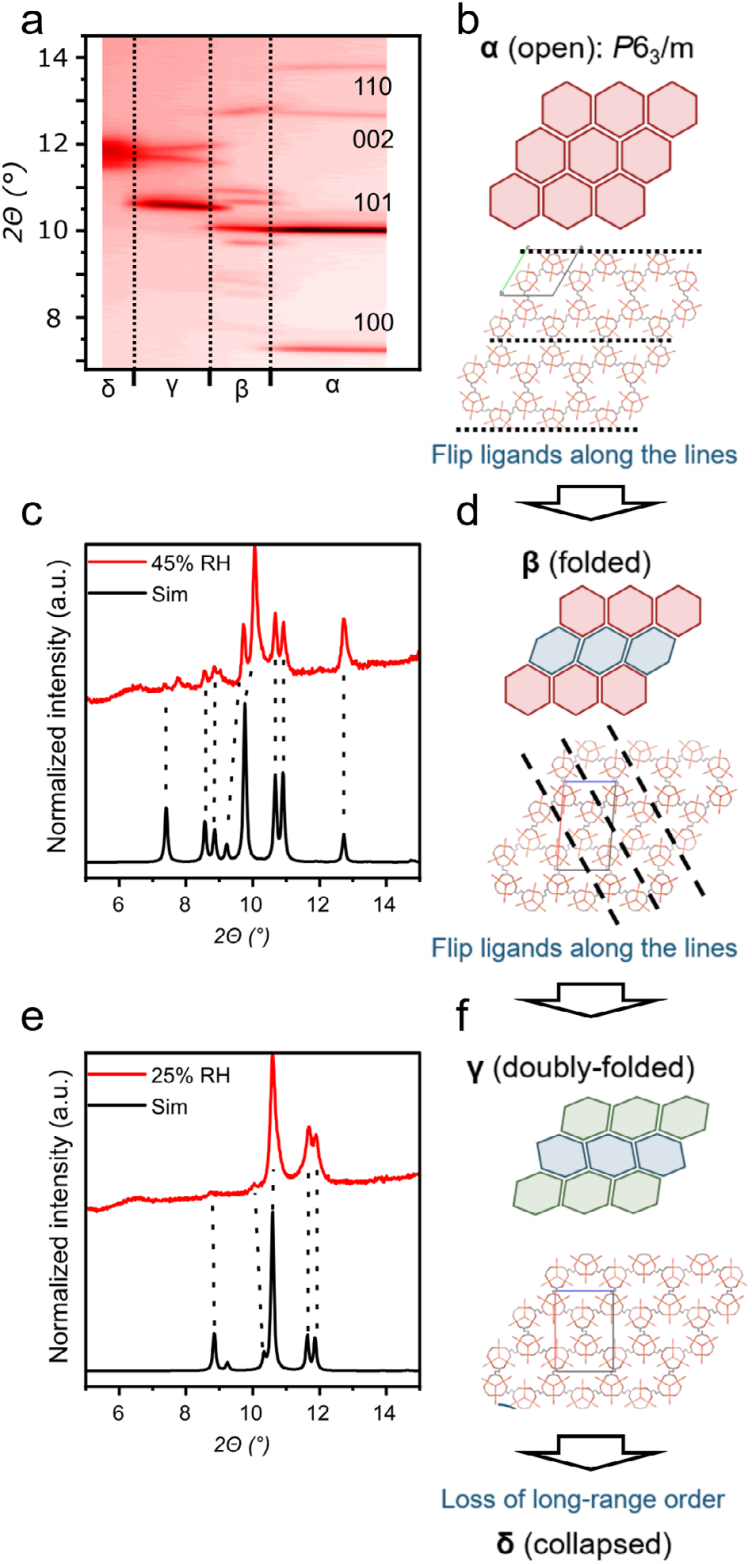
Structural models of the changes of MIL−88 A during the swelling process. a) in situ humidity PXRD pattern (Figure 3) with assigned phases, c,e) shows individual PXRD patterns and the respective simulated patterns derived from the structural models of the α‐, β‐, and γ‐phases depicted in (b,d,f), respectively.

After the structural SCXRD analysis of solvated MIL−88 A, we also analyzed the structural changes during the removal of guest molecules. From the in situ PXRD data of **MIL−88 A Mic** (**Figure** **5**), we were able to identify four different crystallographically independent phases, namely α (open; *P*6_3_/*m*), β (folded), γ (double‐folded), and δ (closed/collapsed). The α phase can be easily compared to the results from the single‐crystal diffraction. The data quality is too unsatisfactory for the other three phases due to fluorescence and mechanical strain in the material during the measurements. We came up with hypothetical structures for the beta and gamma phases (Figure 5). For the first transition (α → β), the linkers along the dotted black line likely undergo a flipping (see Figure 5, dotted black lines), which leads to a deformation of some of the pores (blue tilted hexagons). In the second transition (β → γ), the remaining linkers could also flip their conformation (indicated with dashed black lines). This leads to the green tilted hexagons. The last transition (γ → δ) could be described as a loss of long‐range order. These confirmation changes would explain the changes in the experimental in situ PXRD patterns. Additionally, we simulated PXRD patterns of the β and γ phases, which show an overall similarity with the experimental diffraction patterns (Figure 5). A detailed description and the structure models can be found in the Supporting Information.

## 3 Conclusion 

In this study, we investigated the synthesis of MIL−88 A by different methods, producing large crystals and providing the first single crystal structure of MIL−88 A (Fe). We expanded the study to the synthesis of bulk powders for extended materials characterization using established solvothermal methods in DMF and green synthesis protocols based on hydrothermal and mechanochemical methods. We characterized all samples by PXRD, N_2_ sorption, TGA, SEM, and THz Raman spectroscopy. Additionally, we performed water vapor sorption, in situ humidity PXRD, and optical calorimetry experiments in order to investigate the swelling process and the stability toward water.

We show that the resulting four sets of MIL−88 A samples differ in crystal structure, crystal size distribution, and morphology. Upon drying, we observe a collapse of the structure with large differences in the nature and long‐range order within the series, which is complemented by a large expansion of the lattice by over 80 V%^[^ ^17^ ^]^ upon suspension in water, where all four samples form the same hydrated state. In combination with the in situ data, we developed a hypothetical structural concept that could explain the changes in the XRD patterns during the swelling process. We further probe the capability of water uptake by volumetric water adsorption experiments and observe that the obtained isotherms, in some cases, strongly differ within the series of samples. In addition, all isotherms show multiple steps and hysteresis that we can associate with structural expansion upon water adsorption. We analyzed the structural response of MIL−88 A toward water on water‐suspended samples by PXRD and THz Raman spectroscopy and identified that the water‐filled states of all four samples are comparable. However, by in situ humidity PXRD, we are able to monitor strong differences in the structural response at intermediate humidity.

We identified at least four different crystallographic phases, indicating the formation of well‐defined structures in contrast to an ill‐defined gradual swelling of polymeric materials. However, their presence occurs at different RH levels among the differently synthesized samples, indicating a strong synthesis‐function relationship, which has not been investigated so far. In addition, we demonstrate that THz Raman spectroscopy is a fast, sensitive, and non‐destructive technique for determining the open or closed structure of MIL−88 A, allowing us to probe the process. Of all samples investigated, the mechanochemically synthesized MIL−88 sample (**MIL−88 A Mec**) shows the most facile synthesis procedure paired with the highest water uptake. However, the small crystal size results in a crystallographically ill‐defined transition process, and we assume that the sample is locked in the open pore state. Also, the sample shows the strongest decline upon cycling investigated by in situ calorimetric methods. In the future, we would like to further extend the analysis of the enthalpy changes during the adsorption process to illustrate the utilization of the materials not only as “smart” water harvesters but also toward responsive adsorption‐based chiller technologies.

## Conflict of Interest

The authors declare no conflict of interest.

## Author Contributions

T.M. led the project, synthesized the materials, interpreted the data, and prepared the manuscript. F.H. performed sorption measurements and interpretation. S.R. and D.L. performed INFRAsorp measurements and interpretation. K.G. recorded Raman spectra and interpreted the data. S.B. performed in situ PXRD measurements. S.T. analyzed PXRD data and developed the hypothetical structures. S.C. performed SCXRD analysis and structure refinement. S.K. supervised the research. All authors wrote and commented on the manuscript.

## Supporting information



Supporting Information

## Data Availability

Data supporting this study are contained in the manuscript and supporting information. Raw data is openly available from the repository DaRUS at https://doi.org/10.18419/DARUS‐5009. The reported crystal structure of MIL−88A and related intensity data can be openly accessed via the Cambridge Crystallographic Data Centre website (ccdc.cam.ac.uk), under the deposition number CCDC2388534.
