# Supplementary material for: Elucidating the Structure and the Impact of Synthesis Methods on the Flexibility of the Metal‐Organic Framework MIL‐88 A (Fe) During Water Capture
Source: Small. 2025 Sep 10;21(40):e06833. doi: 10.1002/smll.202506833 (PMC12508726; doi:10.1002/smll.202506833)
Supplement: Supplementary file 1 — Supporting Information [file SMLL-21-e06833-s001.pdf]

NANO · MICRO
small

Supporting Information

for *Small*, DOI 10.1002/smll.202506833

Elucidating the Structure and the Impact of Synthesis Methods on the Flexibility of the Metal-Organic Framework MIL-88 A (Fe) During Water Capture

*Timo Manitz, Fabian Heck, Sri Rezeki, Kristina Gjorgjevikj, Shun Tokuda, Sebastian Bette, Stefano Canossa, Desirée Leistenschneider and Simon Krause**

Supporting Information for

Elucidating the Structure and the Impact of Synthesis Methods on the Flexibility of the Metal-Organic Framework MIL-88 A (Fe) During Water Capture

Timo Manitz,[a,b] Fabian Heck,[a,c] Sri Rezeki,[d] Kristina Gjorgjevikj,[a,b] Shun Tokuda,[a] Sebastian Bette,[a] Stefano Canossa,[e] Desirée Leistenschneider,[d,f] Simon Krause*[a,g]

[a] T. Manitz, F. Heck, K. Gjorgjevikj, S. Tokuda, Dr. S. Bette, Prof. Dr. S. Krause
Nanochemistry Department
Max Planck Institute for Solid State Research
Heisenbergstraße 1, 70569 Stuttgart, Germany

[b] T. Manitz, K. Gjorgjevikj
Department of Chemistry
University of Stuttgart
Paffenwaldring 55, 70569 Stuttgart, Germany

[c] F. Heck
Department of Chemistry
University of Munich (LMU)
Butenandstraße 5-13, 81377 Munich, Germany

[d] S. Rezeki, Dr. D. Leistenschneider
Institute for Technical Chemistry and Environmental Chemistry
Friedrich-Schiller University Jena
Philosophenweg 7a, 07743 Jena, Germany

[e] Dr. S. Canossa
Department of Chemistry and Applied Biosciences
ETH Zürich
Vladimir Prelog Weg 1, 8093 Zurich, Switzerland

[f] Dr. D. Leistenschneider
Center for Energy and Environmental Chemistry Jena (CEEC Jena)
Philosophenweg 7a, 07743 Jena, Germany

[g] Prof. Dr. S. Krause
Institut für Anorganische Chemie II
Universität Ulm
Albert-Einstein-Allee 11, 89081 Ulm, Germany

*Corresponding author e-mail: simon.krause@uni-ulm.de

Contents

Experimental details........3
Chemicals and equipment........3
Synthesis........4
Characterization........5
PXRD........5
In situ PXRD........5
SEM........6
TGA/EGA........6
Sorption........6
Cycling studies (INFRAsorp)........7
Raman Spectroscopy........7
Single-crystal X-ray diffraction analysis of MIL-88 A........7
PXRD data........11
Cycling study........15
TGA-MS........20
In situ XRD........20
Crystal structure modeling of closed MIL-88 A phases........23
SEM/EDX........26
Raman........28
References........31

Experimental details

Chemicals, methods, and equipment

Fumaric acid (CAS: 110-17-8; >99.0%) and Disodium fumarate (CAS: 17013-01-3; >98.0%) were purchased from TCI. Iron(III)chloride hexahydrate (CAS: 10025-77-1; ≥99%) was purchased from Sigma-Aldrich. Iron(III)chloride (CAS: 7705-08-0; 98%, pure, anhydrous) was purchased from Acros Organics. *N,N*-Dimethylformamide (DMF; CAS: 423640010; ≥99.8%) was purchased from VWR. All chemicals were used as received without further purification.

Cultertubes (Soda-lime glass; polypropylene (PP) screwcaps; 16x100 mm; 12 mL; GL 18); Microwave vials (Biotage 2–5 mL; aluminum caps with septa); Al-Heatblock (custom build by in-house mechanical workshop; h: 8.5 cm, d: 14.5 cm; 24 holes for Culturetubes; Figure S 1); Centrifuge (Medifuge™ kleine Tischzentrifuge); Shaker (Fisherbrand™ Microplate Shakers with 4-Place Platform). Heat plate (heidolph MR Hei-Tec), Microwave (Biotage Initiator+).

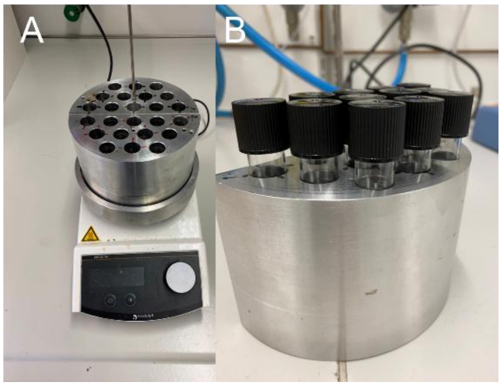

Figure S 1 Custom build Al-heat block.

Synthesis

MIL-88 A was synthesized as previously reported by Chalati *et al*.[1] and Jeong and Lee.[2]

Solvothermal MIL-88 A Sol

Fumaric acid (5 mmol, 580 mg) and iron(III)chloride hexahydrate (5 mmol, 1351 mg) were dissolved in a Schott bottle in DMF (25 mL) at 298 K and agitated at 250 rpm for 5 minutes. The mixture was split equally (5 mL) into 5 culture tubes sealed with polypropylene (PP) screwcaps (Soda-lime glass; 16x100 mm; 12 mL) and the reaction mixture was heated in a custom-made pre-heated Aluminum heat block to 373 K for 24 h. After the reaction had completed (24 h) the tubes were naturally allowed to be cooled to room temperature, the orange powder product was separated from the reaction mixture by centrifugation (4900 rpm, 5 min). Products from the individual reaction tubes were combined and washed three times with fresh DMF followed by centrifugation.

Hydrothermal MIL-88 A Hyd

Fumaric acid (5 mmol; 580 mg) and iron(III)chloride hexahydrate (5 mmol; 1352 mg) were dissolved in a Schott bottle in water (25 mL) at 298 K and agitated at 250 rpm for 5 minutes. The mixture was split equally (5 mL) into 5 culture tubes sealed with polypropylene (PP) screwcaps (Soda-lime glass; 16x100 mm; 12 mL) and the reaction mixture was heated in a custom-made pre-heated Aluminum heat block to 373 K for 24 h. After the reaction had completed (24 h) the tubes were naturally allowed to be cooled to room temperature, the orange powder product was separated from the reaction mixture by centrifugation (4900 rpm, 5 min). Products from the individual reaction tubes were combined and washed three times with fresh water followed by centrifugation.

Microwave MIL-88 A Mic

Fumaric acid (5 mmol, 580 mg) and iron(III)chloride hexahydrate (5 mmol, 1352 mg) were dissolved in a Schott bottle in DMF (25 mL) at 298 K and agitated at 250 rpm for 5 minutes. The mixture was split equally (5 mL) into 5 microwave vials (2-5 mL) and the reaction mixture was heated for one hour to 373 K in a microwave (Biotage Initiator+). After the reaction had completed (1 h) the tubes were naturally allowed to be cooled to room temperature, the orange powder product was separated from the reaction mixture by centrifugation (4900 rpm, 5 min). Products from the individual reaction tubes were combined and washed three times with fresh DMF followed by centrifugation.

Mechanochemistry MIL-88 A Mec
Disodiumfumerate (1 mmol, 160 mg) and iron(III)chloride hexahydrate (1 mmol, 270 mg) were placed in an agate mortar (d: 10 cm) and ground by hand for 10 min. The orange powder product was collected, filled in a culture tube sealed with a polypropylene (PP) screwcap, and washed twice with fresh water followed by centrifugation.

Single crystal synthesis
Single crystals were synthesized in a solvothermal reaction. Fumaric acid (0.08 mmol, 9.6 mg) and iron(III)chloride (0.08 mmol, 13.9 mg) were dissolved in DMF (5 mL). The reaction mixture was placed in a culture tube sealed with a polypropylene (PP) screwcap (Soda-lime glass; 16x100 mm; 12 mL) and was heated in a preheated oven to 393 K for 48 h. After the reaction had completed (48 h) the tubes were naturally allowed to be cooled to room temperature, and the dark red crystals were washed three times with fresh DMF and stored in DMF at room temperature.

Solvent removal and drying (activation)
For activation, most of the washing solvent was removed with a pipette. The reaction tube with the remaining suspension inside was connected to a Schlenk line and heated in a pre-heated aluminum heat block to 373 K. The sample was allowed to equilibrate for 5 min after which the glass vial was slowly evacuated under a dynamic vacuum ($p<10^{-4}$ kPa). The vacuum and temperature was sustained for at least 12 h. The reaction tube was refilled with air and the samples were stored at ambient conditions for characterization.

Characterization

PXRD
Powder X-ray diffraction (PXRD) experiments were performed on a Stoe Stadi P diffractometer (Cu-Kα1, Ge(111)) in Debey-Scherrer geometry. The samples were measured in sealed glass capillaries (OD=0.5/0.7 mm) and spun for better particle statistics. Each pattern was collected in a 2Θ range of 2 to 50°.

In situ PXRD
PXRD patterns under a humidity-controlled atmosphere were collected using a Bruker D8 advanced diffractometer using Cu-Kα1 radiation from a Johann-type Ge111 monochromator and a Lynx Eye detector (Bruker) equipped with a humidity chamber (Anton Paar). The humidity within the chamber was adjusted by mixing a dry and a water-saturated nitrogen stream. A total flow rate of 500 mL/min was applied and the chamber temperature was constantly kept at 298 ± 0.2 K.

SEM

A Merlin (Zeiss) scanning electron microscope (SEM) was used to obtain secondary electron (SE) and energy and angle-selective backscattered electron (EsB) images. The applied voltage was 1.5 kV. The particle size distributions were measured with the software ImageJ.

TGA/EGA

To quantify the amount of solvent in MIL-88 A, thermogravimetric analysis (TGA) was performed. Furthermore, evolved gas analysis (EGA) was performed to identify the compounds released during TG analysis. Prior to the TGA/EGA measurement, blank samples were measured to minimize buoyancy effects in the TGA and to obtain background spectra in the EGA. For the TGA measurement, about 10 mg of MIL-88 A powder sample was loaded into an Al_2O_3 crucible and subsequently transferred into the furnace chamber. The TGA was carefully purged several times with helium to avoid atmospheric contaminates. The sample was heated with a heating rate of 10 K min^{-1} to 1073 K (Netzsch Jupiter STA 449 F3, SiC furnace, type S thermocouple sample carrier, 70 mL min^{-1} He (5N Air Liquid) stream). The TGA data were analyzed with the software Proteus Analysis (Netzsch). Sampling of the evolved exhaust gas was performed at 323, 433, 573, 698, 813 or 863, and 923 or 973 K. The sampled gas was transferred via an inert, heated line to a gas chromatograph (GC, Agilent 8890 GC System, HP-5MS UI column with 30 m × 0.25 mm × 0.25 µm, 5:1 injection split ratio, sample inlet temperature 573 K, He carrier with 1.2 mL min^{-1}). The GC oven was heated from 323 to 473 K with a heating rate of 20 K min^{-1}. After passing the GC, the separated components were detected in a mass spectrometer (MS, Agilent 5977B GC/MSD, EI mode, 503 K ion source temperature, 423 K quadrupole temperature). The total ion current of the GC peaks was measured. The mass spectra were analyzed by means of best-matching database entries (NIST database, MSD ChemStation Data Analysis).

Sorption

Gas adsorption experiments for the MOF samples were performed on a Quantachrome Instruments Autosorb iQ MP with nitrogen at 77 K. Vapor adsorption experiments for the presented materials were performed on a Quantachrome Instruments Autosorb iQ MP device with water at 298 K. The samples were degassed for 12 h at 393 K under vacuum prior to the adsorption studies. Values of the adsorbed amount V_{STP} [cm^3 g^{-1}] were converted to gravimetric amount adsorbed per gram of material [g g^{-1}] = V_{STP}/22414 cm^3/mol * 18.015 g/mol.

Cycling studies (INFRAsorp)

Optical calorimetry measurements were conducted utilizing the INFRAsorp instrument (Fraunhofer IWS, Dresden). Every measurement was performed at 1.0 bar and 298 K. Prior to each measurement, all samples were subjected to preheating at 120 °C under vacuum for 12 h. Upon cooling to ambient temperature, approximately 10 mg of the sample was positioned in the sample holder. The sample was then transferred to the instrument and purged with a dry nitrogen flow for 600 s. A nitrogen flow, saturated with water vapor at a rate of 50 mL/min, was directed through the adsorbent for 1200 s, during which the temperature response of the adsorption process was documented. With water saturated nitrogen was produced by passing nitrogen through a sealed water reservoir at ambient temperature before being supplied to the INFRAsorp apparatus. The humidity was measured with a HYT939 sensor. The interval between the adsorption and desorption cycles has been established at 120 s. The experiment was conducted at varying relative humidity levels of 20%, 30%, 55%, 70%, and 90%. Additionally, 100 adsorption and desorption cycles were conducted to evaluate thermal stability with a relative humidity of 90%.

Raman Spectroscopy

Raman spectra were recorded on a home-built system with a 785 nm laser equipped with a Raman Probe (TR-Probe from Coherent with a 300 mW power at the sample port with a steerable non-contact optics) and connected to a spectrograph (Andor Kymera 328i, with an Andor iDUS 420 CCD detector). The measurements were carried out in sealed quartz capillaries with a 0.7 mm diameter, without further sample preparation. The laser power on the sample was kept at 40% and no significant sample degradation was detected. All measurements were carried out at room temperature. The analysis of the data was done by SpectraGryph 1.2 as well as OriginPro 2022b. All spectra shown have been processed by removal of cosmic rays, their background has been subtracted, they have been smoothed using the Savitsky-Golay filter and normalized to the peak with the highest intensity. This has not affected signal disposition and all conclusions are valid to the raw data as well which is provided with the SI.

Single-crystal X-ray diffraction analysis of MIL-88 A

Single-crystal X-ray diffraction data for MIL-88 A (kept in *N,N*-dimethylformamide (DMF)) were collected at the Bessy Synchrotron facility of the Helmholtz Zentrum Berlin (Beamline 14.2). A crystal was isolated and mounted on a MiTeGen loop in a droplet of a NVH cryoprotectant oil. Diffraction data were acquired using a monochromatic 0.79999 Å radiation at a temperature of 100 K, using a cold dry nitrogen stream produced with an Oxford Cryostream (Oxford Cryosystems Ltd., Oxford, United Kingdom). The crystal was placed directly under the

Cryostream at 100 K without any temperature ramp. Frames were collected on a Pilatus3S 2M, running a phi scan with oscillation of 0.2° and covering an angular range of 360°. The resulting data were processed using the software CrysAlisPro ver. 43.90 (Rigaku Oxford Diffraction, (2017), CrysAlisPro Software System, Version 1.171.40.67a, Oxford, UK), which included indexing, intensities integration and empirical absorption correction. As can be seen from the following reciprocal space reconstructions of the main planes, the crystal is fractured in several components with a major domain. Indexing of a multicrystal failed in producing suitable reflection files for a satisfactory indexing, while by integrating the data as one single domain and adding a 2x multiplier to the integration box automatically generated by CrysAlisPro resulted in an acceptable list of intensities with an overall Rint of 7.24 at 0.75 Å resolution.

Structure solution and refinement were conducted by using the software suite Olex2 v1.5,[3] using the programs ShelXT (intrinsic phasing) and ShelXL (least squares) respectively.[4] A DMF molecule was observed in the residual electron densities, coordinated to Fe atoms. Ill-behaved displacement parameters and inconsistent trends between those of the coordinated oxygen and of the remaining atoms of the DMF led to the hypothesis that this open metal site could be partly occupied by a water molecule, and partly by a DMF molecule. Therefore, all atoms of the DMF molecule except the oxygen were given free occupancy, which refined to a value of 0.399(15), whereas the oxygen was given full occupancy. All atoms in the asymmetric unit have been refined with anisotropic displacement parameters, with the exception of hydrogen atoms. These have been fixed in idealized positions by AFIX43 constraints, except those belonging to the methyl groups of the partly occupied DMF, which has been fixed to avoid refinement instability linked to their rotation around the nitrogen-carbon bond.

Below, the main planes of reciprocal space reconstructed from the data are displayed, together with the refined asymmetric unit of MIL-88 A; ellipsoids are set to show a 30% probability for the sake of clarity.

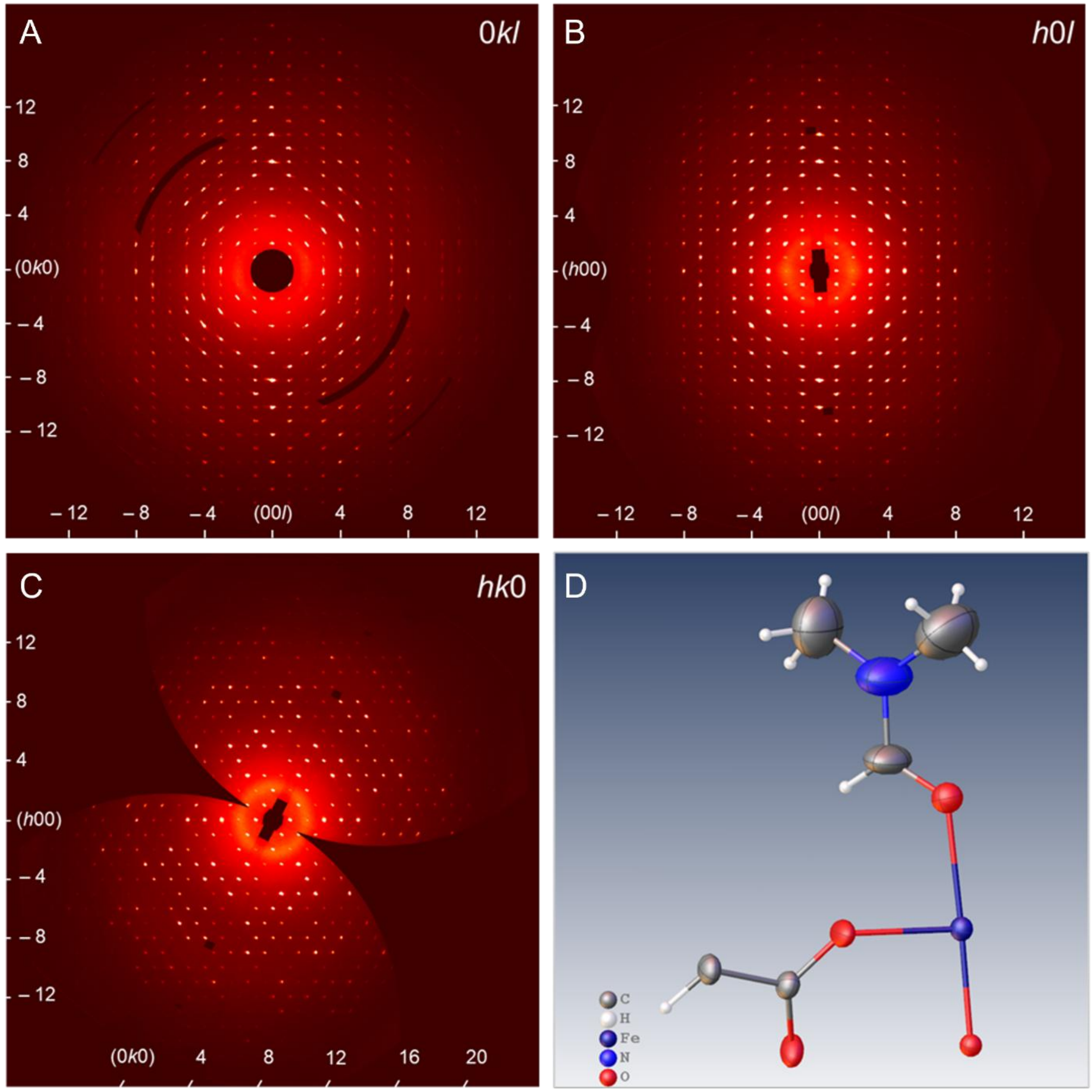

Figure S 2 Diffraction patterns and refined asymmetric unit for MIL-88 A (DMF).

Table S 1 Crystal data and structure refinement for MIL-88 A (DMF).

Empirical formula	$C_{20.1}H_{33.3}Fe_3N_{2.7}O_{17.5}$
Formula weight	760.34
Temperature/K	100
Crystal system	hexagonal
Space group	$P6_3/m$
a/Å	12.6796(4)
b/Å	12.6796(4)
c/Å	13.7147(3)
α/°	90
β/°	90
γ/°	120
Volume/Å^3	1909.54(13)
Z	2
ρ_{calc}g/cm^3	1.322
μ/mm^{-1}	1.651
F(000)	782.0
Crystal size/mm^3	0.07 × 0.03 × 0.03
Radiation	synchrotron (λ = 0.79999)
2Θ range for data collection/°	5.35 to 64.36
Index ranges	-14 ≤ h ≤ 14, -16 ≤ k ≤ 16, -18 ≤ l ≤ 18
Reflections collected	27204
Independent reflections	1637 [R_{int} = 0.0724, R_{sigma} = 0.0214]
Data/restraints/parameters	1637/33/76
Goodness-of-fit on F^2	1.126
Final R indexes [I>=2σ (I)]	R_1 = 0.0537, wR_2 = 0.1836
Final R indexes [all data]	R_1 = 0.0584, wR_2 = 0.1880
Largest diff. peak/hole / e Å^{-3}	0.89/-0.51

PXRD data

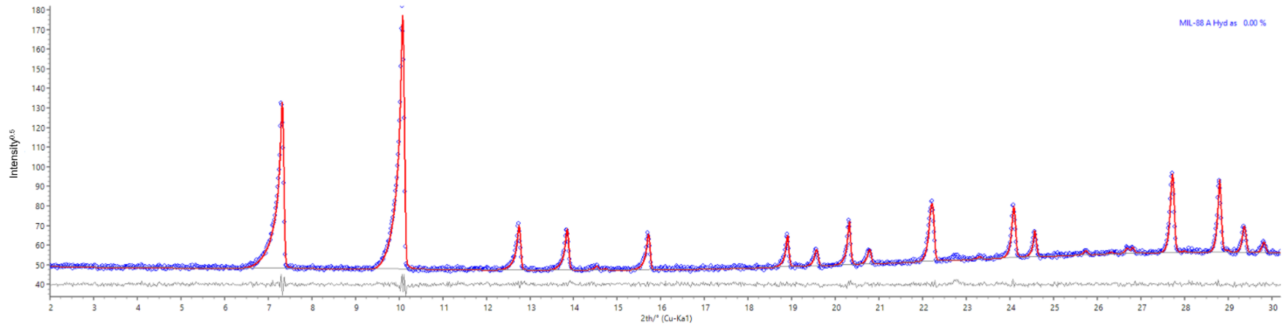

Figure S 3 Pawley refinement of MIL-88 A Hyd as.

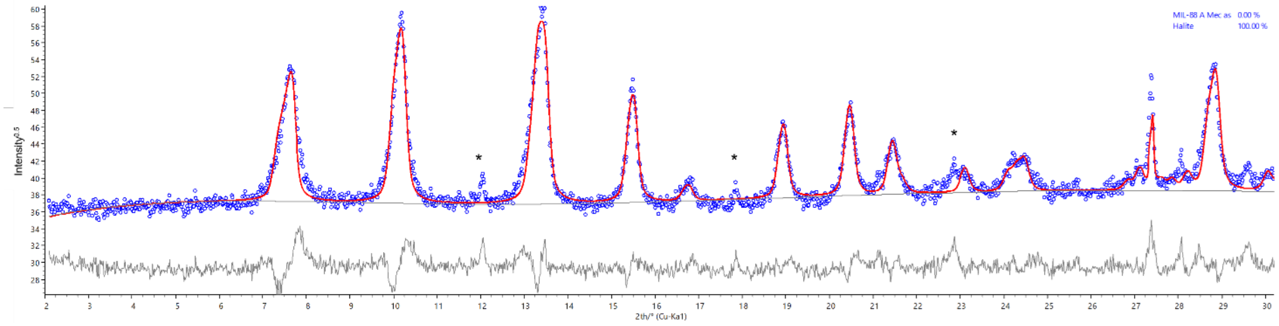

*Figure S 4 Pawley refinement of MIL-88 A Mec as. * marks unindexed reflexes, which are assigned to different phases, not related to the linker, metal node or other known phases.*

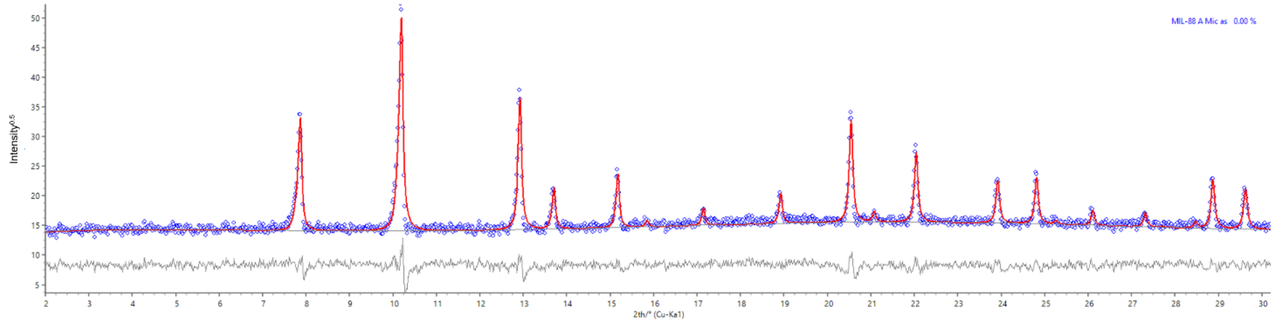

Figure S 5 Pawley refinement of MIL-88 A Mic as.

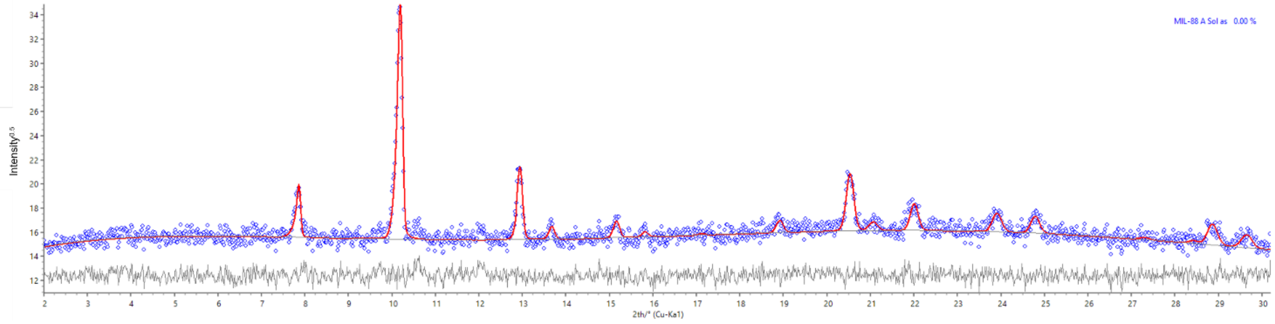

Figure S 6 Pawley refinement of MIL-88 A Sol as.

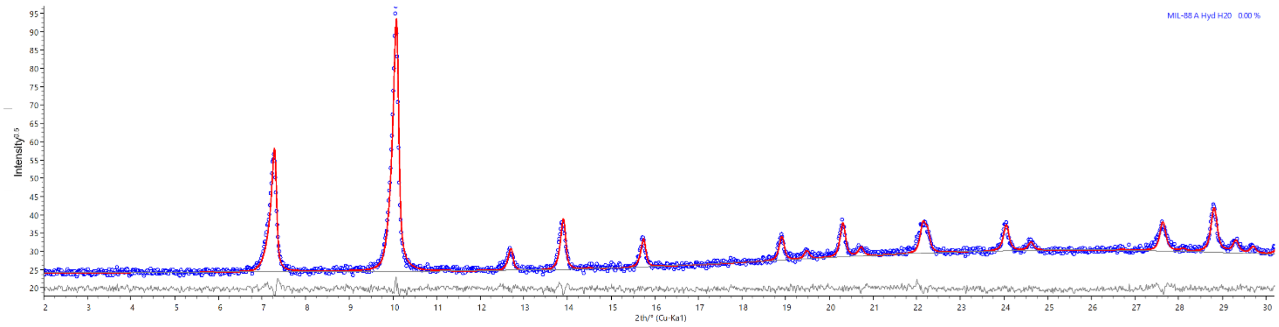

Figure S 7 Pawley refinement of MIL-88 A Hyd H_2O.

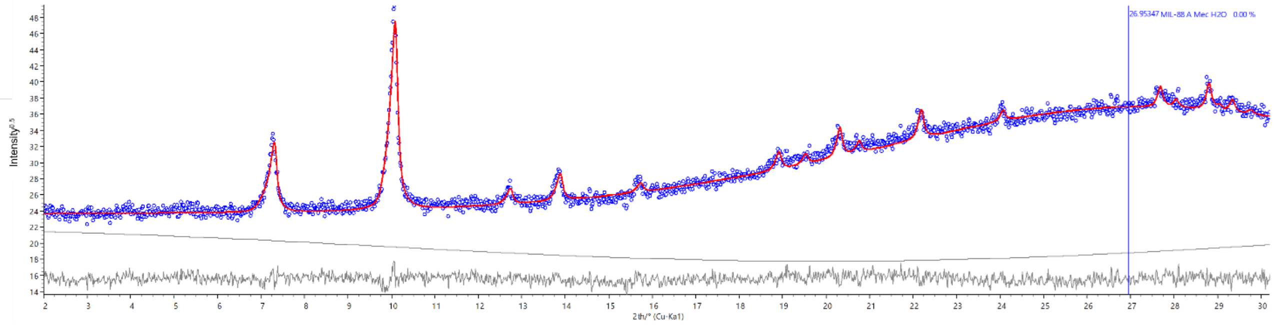

Figure S 8 Pawley refinement of MIL-88 A Mec H_2O. Solvent hump with additional peaks at position 26.95° and 41.36° refined.

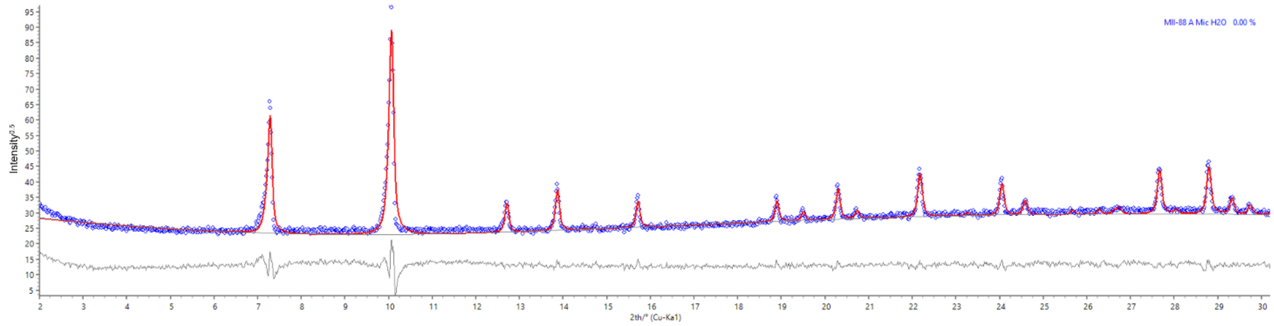

Figure S 9 Pawley refinement of MIL-88 A Mic H_2O.

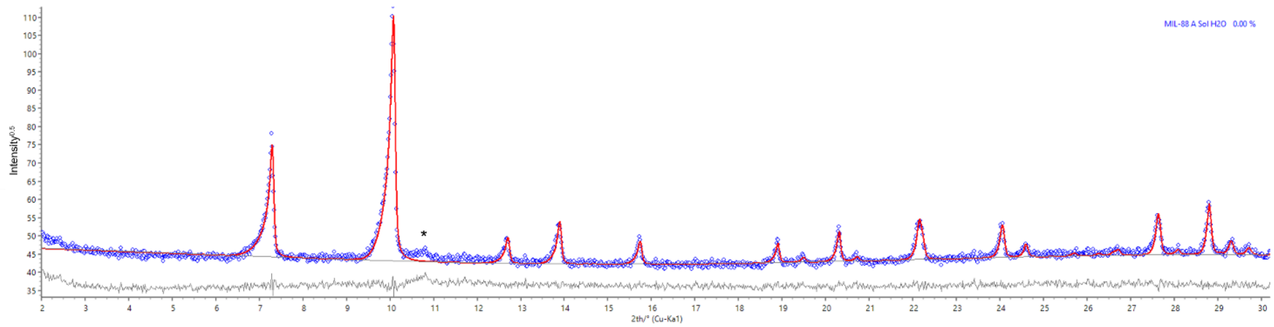

*Figure S 10 Pawley refinement of MIL-88 A Sol H_2O. * marks unindexed reflexes, which are assigned to different phases, not related to the linker, metal node or other known phases.*

Table S 2 Synthesis conditions of MIL-88 A and the respective crystallographic analysis and crystal size distribution.

Synthesis method	Solvothermal	Hydrothermal	Microwave	Mechano
Sample code	MIL-88 A Sol	MIL-88 A Hyd	MIL-88 A Mic	MIL-88 A Mec
Reaction time (h)	24	24	1	0.16 (10 min)
Reaction temperature (K)	373	373	373	298
Solvent	DMF	Water	DMF	Solvent-free
Lattice parameter as Pawley (Å; 298 K)	a=12.86(1)<br>c=13.58(1)	a=13.79(1)<br>c=12.70(1)	a=12.84(1)<br>b=13.60(1)	a=13.32(1)<br>c=13.15(1)
Unit cell Volume as made (Å^3; 298 K)	1947(2)	2094(1)	1940(1)	2016(1)
Rwp	5.651	6.643	7.947	4.667
Lattice parameter water Pawley (Å; 298 K)	a=13.84(1)<br>c=12.67(1)	a=13.85(1)<br>c=12.66(1)	a=13.83(1)<br>c=12.68(1)	a=13.81(1)<br>c=12.67(1)
Unit cell Volume water (Å^3; 298 K)	2102(1)	2102(1)	2100(1)	2100(2)
Rwp	2.711	3.841	5.714	6.879
Space group	$P6_3/m$	$P6_3/m$	$P6_3/m$	$P6_3/m$
Mean Crystal length (µm)	3.87 ± 1.53	4.20 ± 3.66	1.99 ± 0.93	non quantifiable
Mean Crystal width (µm)	3.37 ± 1.26	1.34 ± 0.65	1.01 ± 0.51	non quantifiable

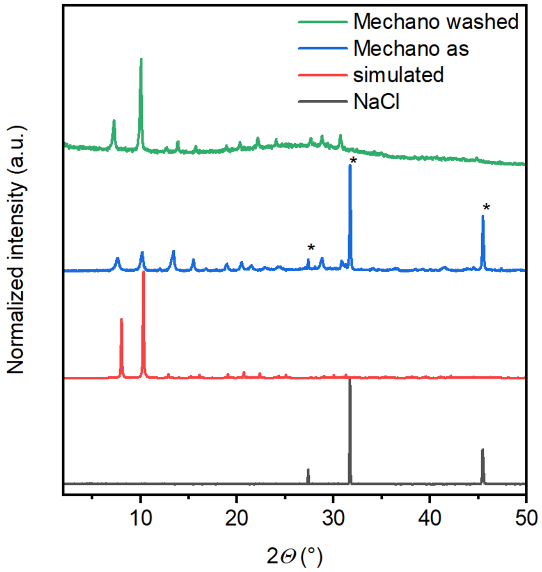

Figure S 11 PXRD patterns of NaCl (black), MIL-88 A Mec after synthesis (as, blue), after washing three times with water (washed, green) and the simulated PXRD pattern from the MIL-88 A single crystal structure (simulated red).

Cycling study

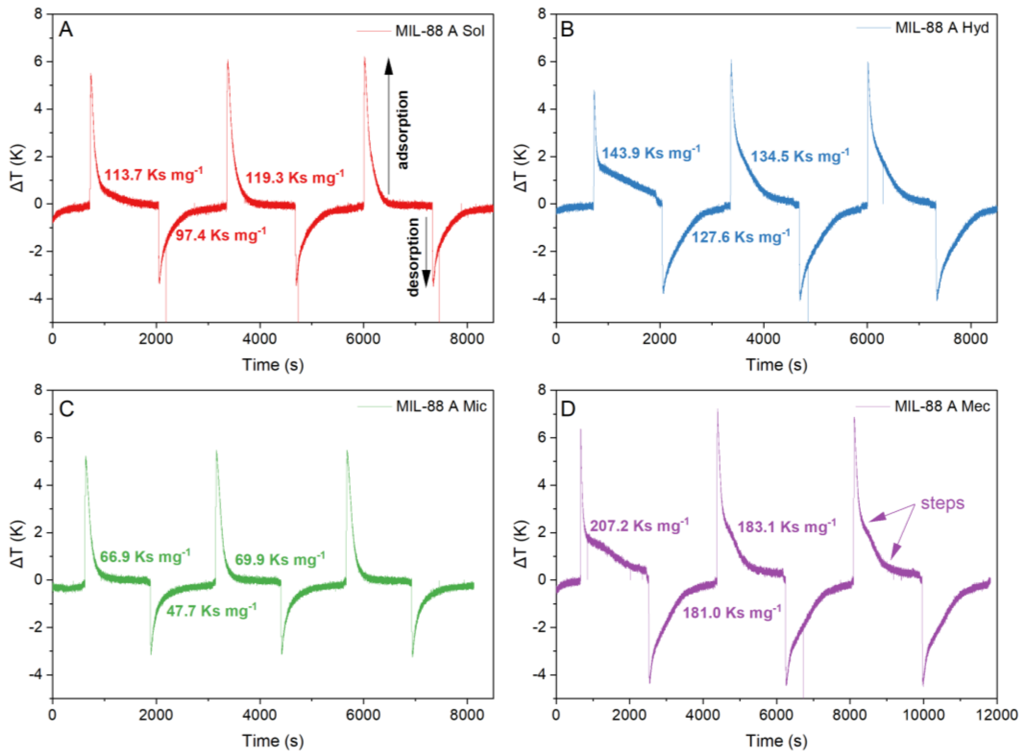

Figure S 12 Water adsorption measurement for 3 cycles of adsorption and desorption at 90% RH for (A) MIL-88 A Sol, (B) MIL-88 A Hyd, (C) MIL-88 A Mic, and (D) MIL-88 A Mec.

The peak of MIL-88 A Sol is relatively similar to the peak of MIL-88 A Mic, which is sharp and narrow. Meanwhile, the peak shape of MIL-88 A Hyd is relatively the same as MIL-88 A Mec, which is broad and does not reach the baseline after the adsorption time. Apparently, MIL-88 A Mec and MIL-88 A Hyd exhibit a comparable water adsorption process as also the subsequent cycles, as well as the desorption cycles, show the same peak shape for the two materials. Both of these materials show a broader particle size distribution and generally larger particles. Thus, it is assumed that this mainly affects heat formation and dissipation during the adsorption and desorption processes. For all materials, the desorption peak areas are smaller than the adsorption peak areas. This can either point to an incomplete desorption due to strong water-adsorbent interaction, or heat dissipation due to a kinetically limited process.

Furthermore, it should be noted that INFRAsorp was not employed as an absolute calorimetry method[5] in this study. Rather, its primary objective was to demonstrate the cycle stability of MIL-88 A during water adsorption and desorption. It is noteworthy that the peak areas of the

samples MIL-88 A Hyd and Mic differ, despite the similarity in their water uptake in vapor sorption experiments. In the context of INFRAsorp measurements, the heat change is significantly influenced by several factors, including the size of the particles, the packing density of the sample bed, the heat capacity of the bed, and the distribution of the particles. This phenomenon can be further influenced by the anisotropy of the needle-shaped particles of MIL-88 A.

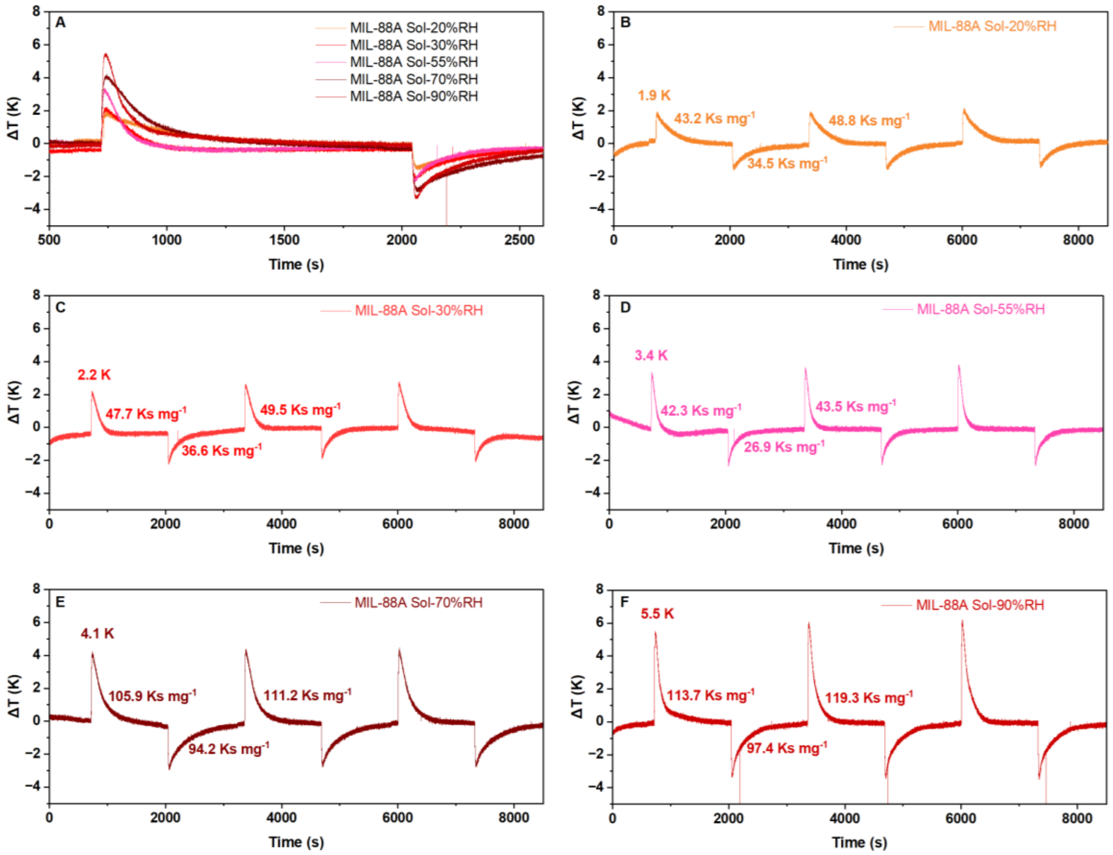

Figure S 13 Infrasorp measurement for MIL-88 A Sol in different relative humidity.

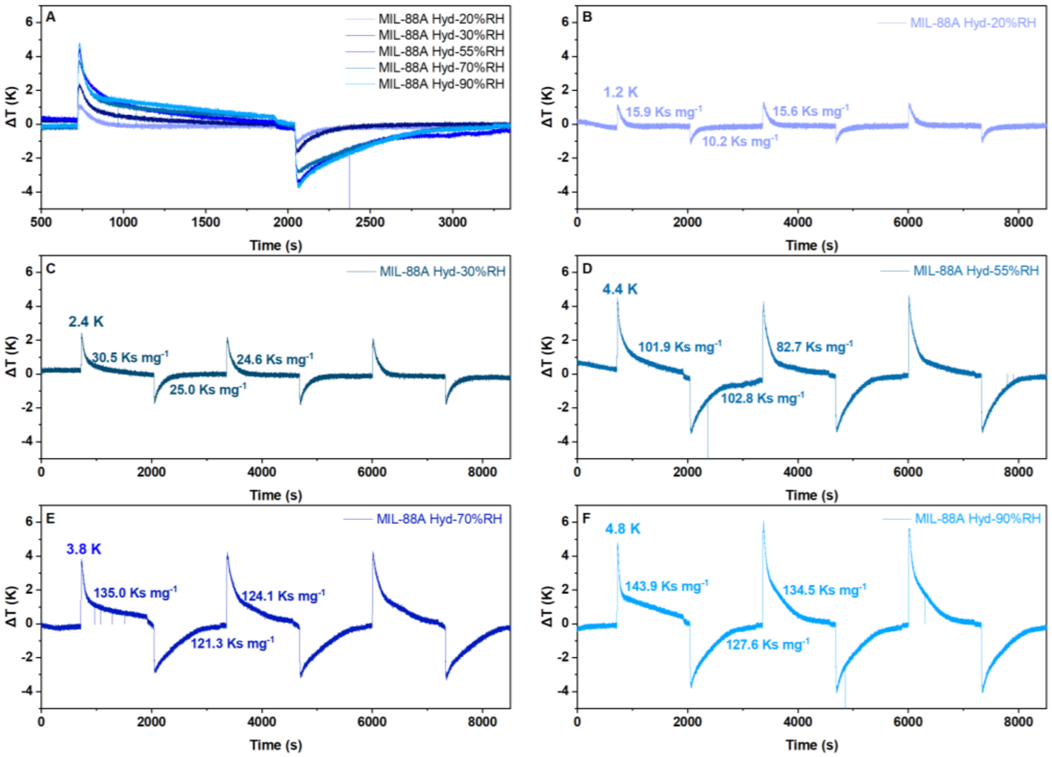

Figure S 14 Infrasorp measurement for MIL-88 A Hyd in different relative humidity.

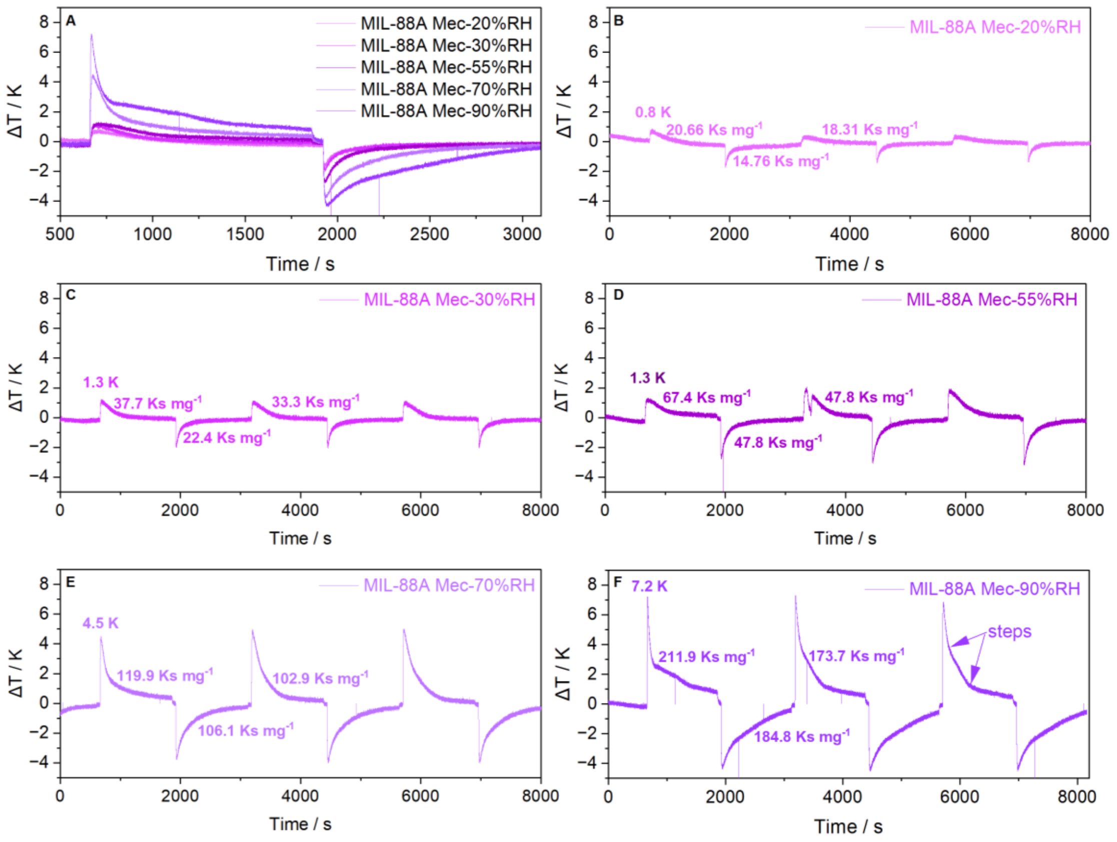

Figure S 15 Infrasorp measurement for MIL-88 A Mec in different relative humidity.

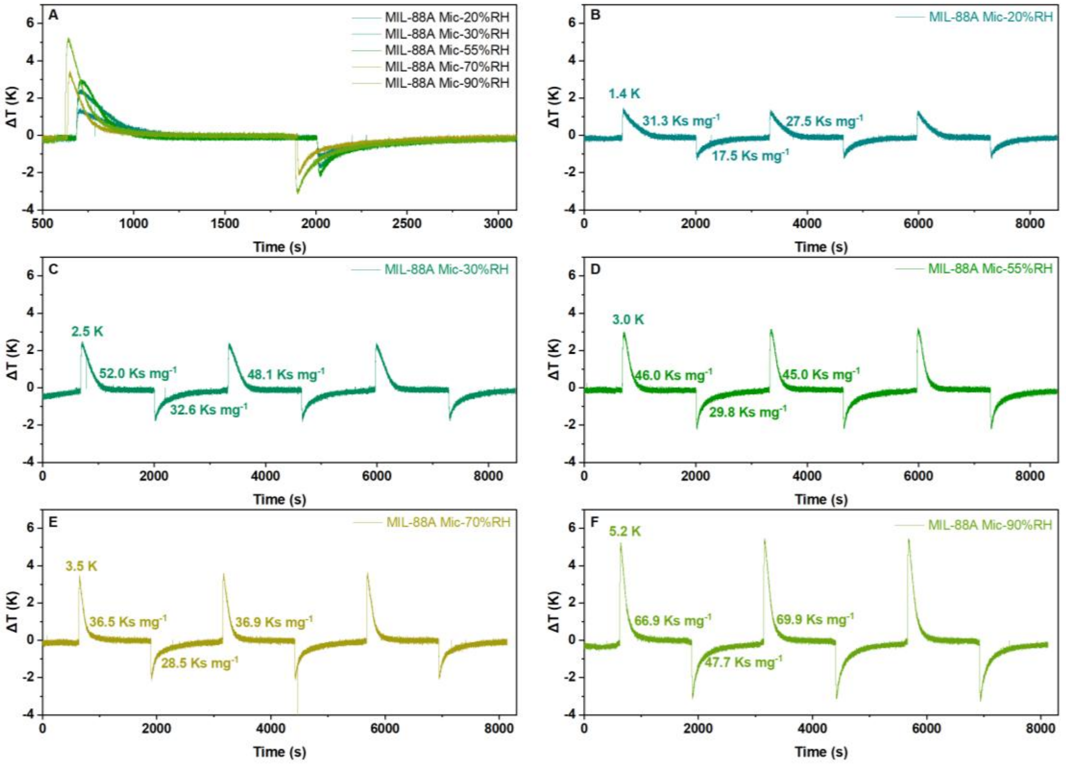

Figure S 16 Infrasorp measurement for MIL-88 A Mic in different relative humidity.

TGA-MS

The TGA analysis presented herein was conducted on the as-made samples to evaluate the necessity of washing. The removal of unreacted linkers was achieved through the implementation of a three-step washing process, employing the reaction solvent to remove any residual linkers before proceeding with further analysis.

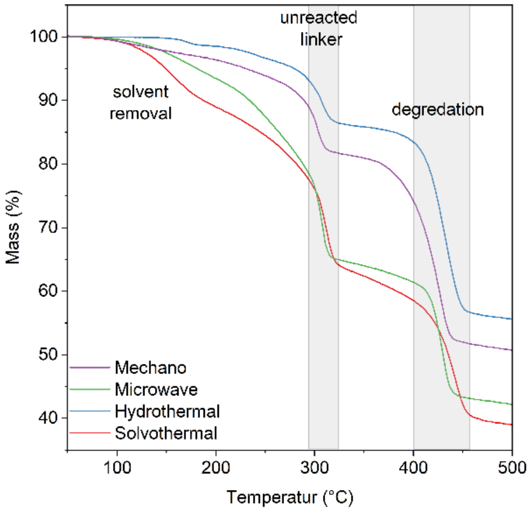

Figure S 17 TGA curves (25-500 °C) of MIL-88 A synthesized via mechanical (purple), microwave (green), hydrothermal (blue), and solvothermal (red) synthesis approaches.

In situ XRD

Since all samples contain iron, all XRD patterns recorded with Cu K_α show a high background due to fluorescence. Additionally, it is worth mentioning that the samples change mechanically during the process. Due to the water absorption, the sample bed will swell and break during desorption.

For the samples MIL-88 A Hyd and Mec, we noticed a shift in the PXRD by 2° *2Θ.* This baseline shift was corrected manually before the data was analyzed.

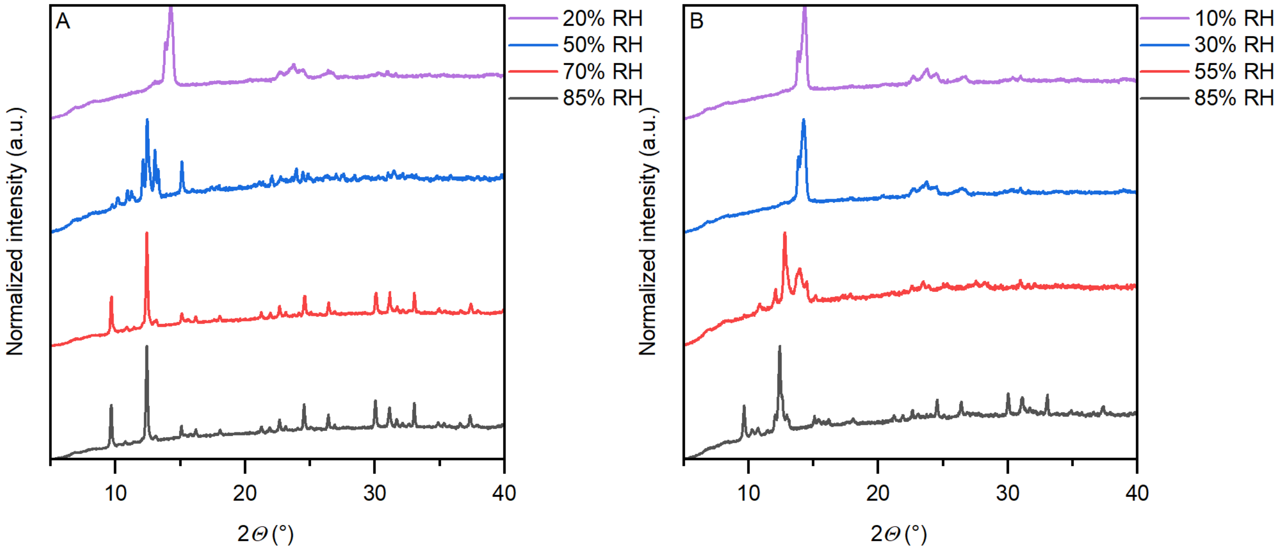

Figure S 18 Selected PXRD pattern during desorption (A) and adsorption (B) process from in situ humidity measurements of MIL-88 A Hyd.

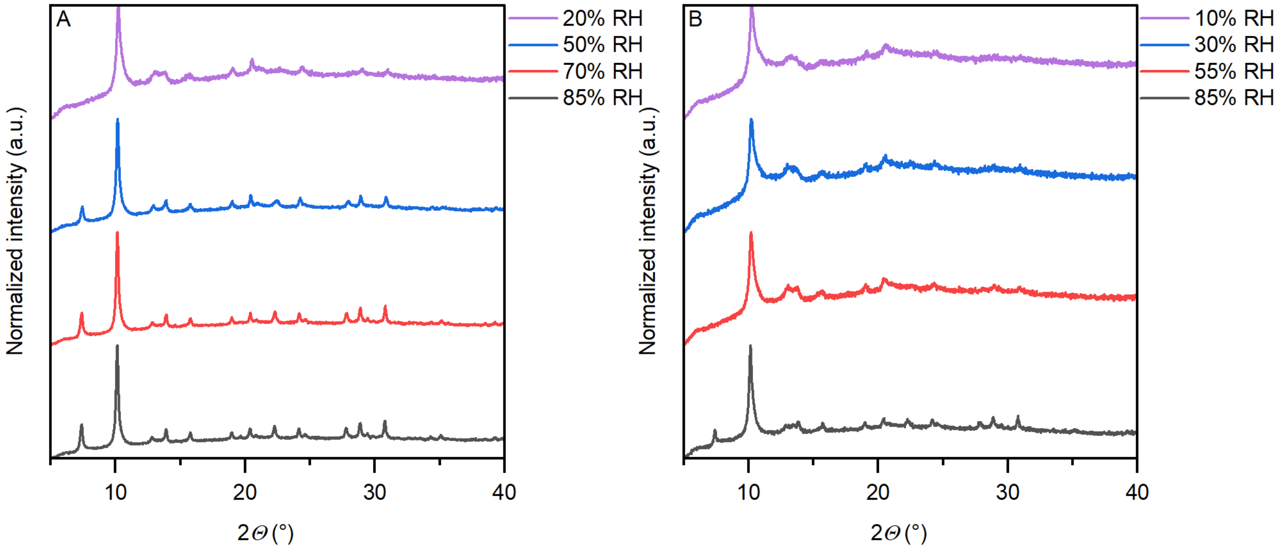

Figure S 19 Selected PXRD pattern during desorption (A) and adsorption (B) process from in situ humidity measurements of MIL-88 A Mec.

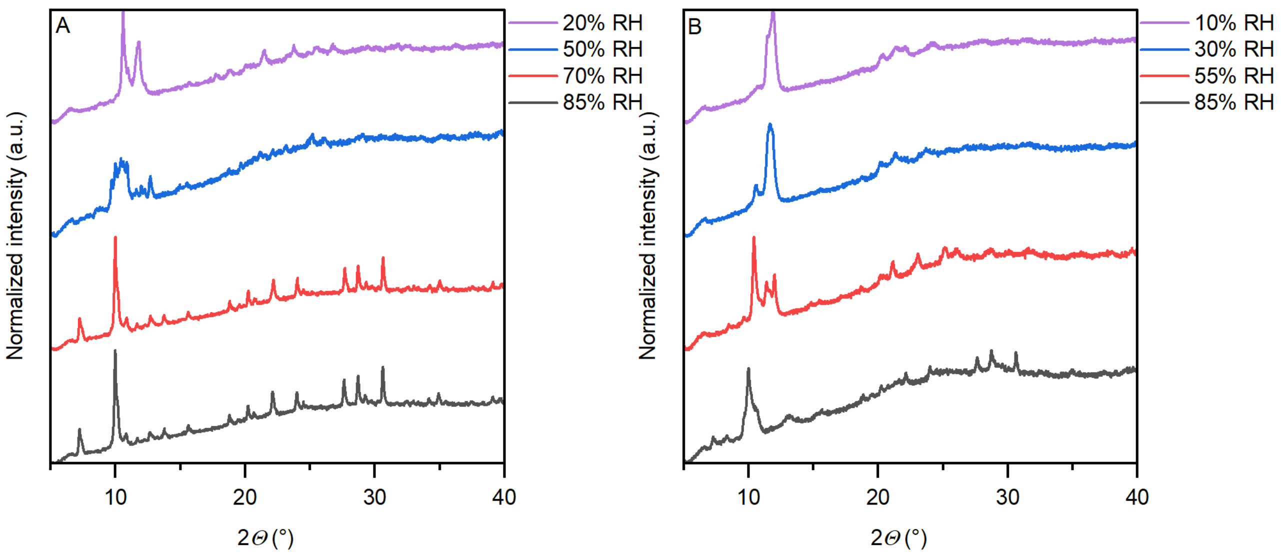

Figure S 20 Selected PXRD pattern during desorption (A) and adsorption (B) process from in situ humidity measurements of MIL-88 A Sol.

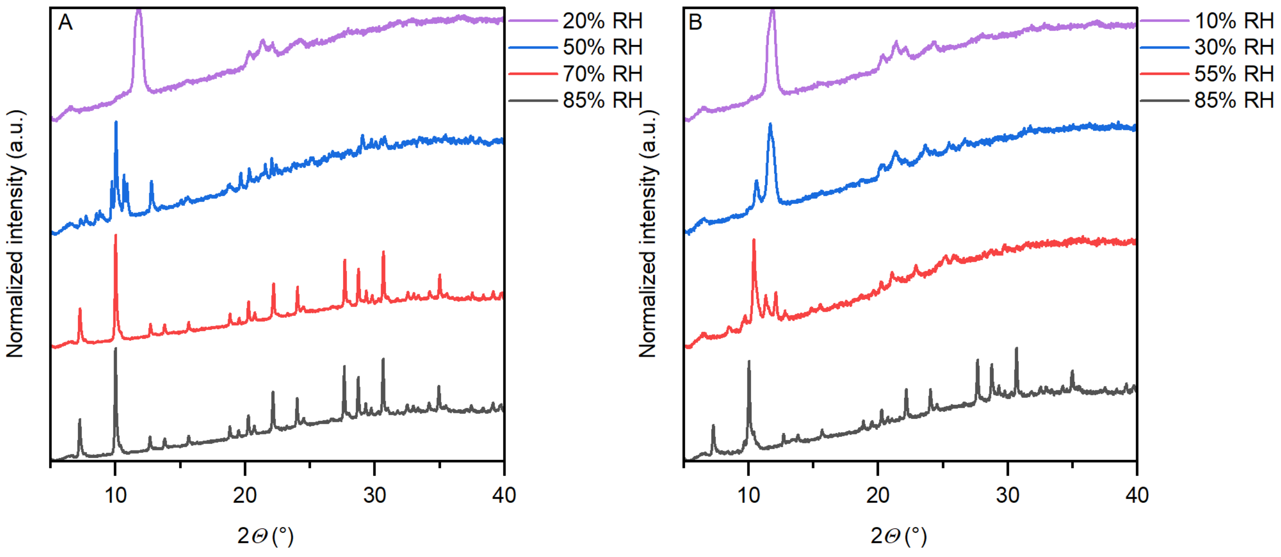

Figure S 21 Selected PXRD pattern during desorption (A) and adsorption (B) process from in situ humidity measurements of MIL-88 A Mic.

Crystal structure modeling of closed MIL-88 A phases

The possible conformational isomers of MIL-88 A were modeled by considering the honeycomb tiling of hexagons with three possible angles (Figure S 22). As it was evident from the observed crystal structure of MIL-88 A in the “open” solvated phase (CCDC 2388534), the Fe_3O nodes were found to be tilted by ω = 8.543º from the direction of the neighbor Fe_3O node (Figure S 22 A). This tilted node orientation originates from the characteristic zigzag-shaped geometry and the coplanar conformation of the fumarate linkers. Although all fumarate linkers show the same conformation in the observed crystal structure and thus all nodes show the C_3-symmetric connectivity, one can also consider the inverted conformation of fumarate to generate different node environments (Figure S 22 B). Note that the possible inter-node angle is either 120º, 120º + 2ω, or 120º - 2ω. Then, by connecting these three angles with the fixed length of linkers (edges), five kinds of hexagons can be drawn as possible partial structures of MIL-88 A (Figure S 22 C). The full MIL-88 A structure is represented by the honeycomb tiling of these hexagons (Figure S 22 D). The atomistic structural models of potential MIL-88 A structures were first modeled with the unit cell parameters calculated from ω = 8.543º and the edge length of 7.32 Å, and the validity of the tiling model was checked by comparing the simulated PXRD patterns with the experimental observations. This screening process identified the models β and γ as highly possible candidates for the observed β and γ phases, respectively, and rejected the possibility of the other three tested models. Finally, each structural model for β and γ phases was geometrically optimized with the Forcite module of Material Studio suite. The develop structure models are shown in Figure S 23-Figure S 25.

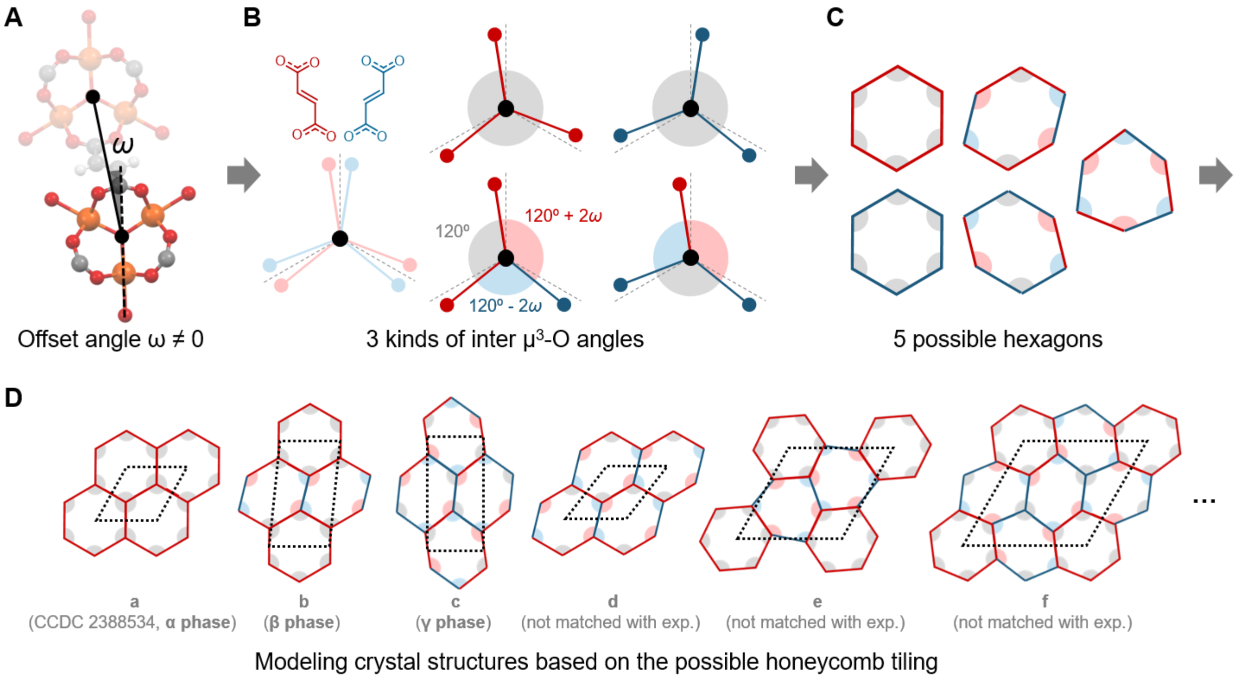

Figure S 22 Procedures for modelling the closed MIL-88 A phases.

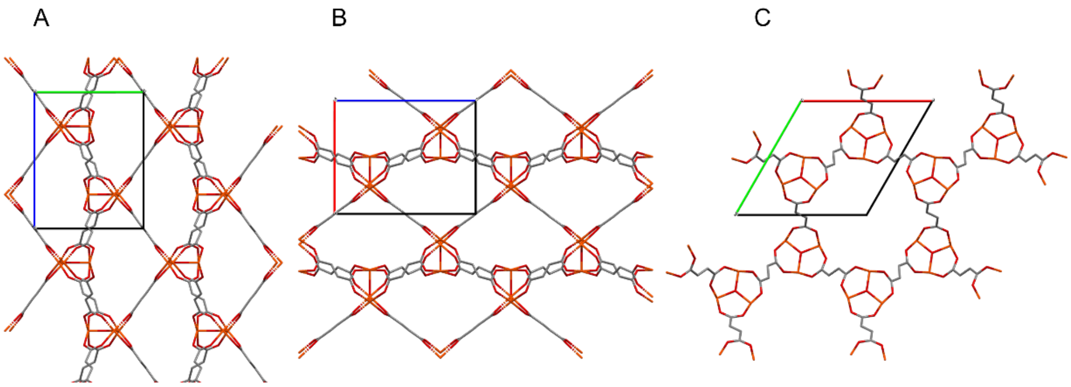

Figure S 23 Single crystal structure of the α phase of MIL-88 A.

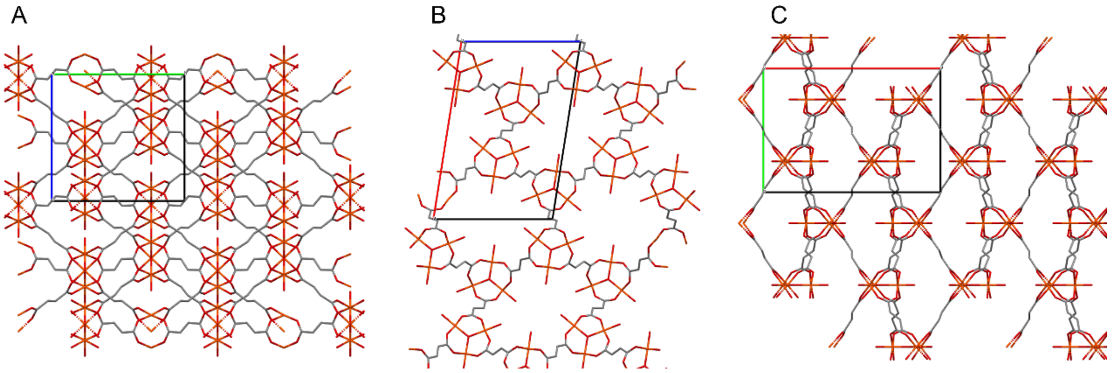

Figure S 24 Modeled structure of the β phase of MIL-88 A.

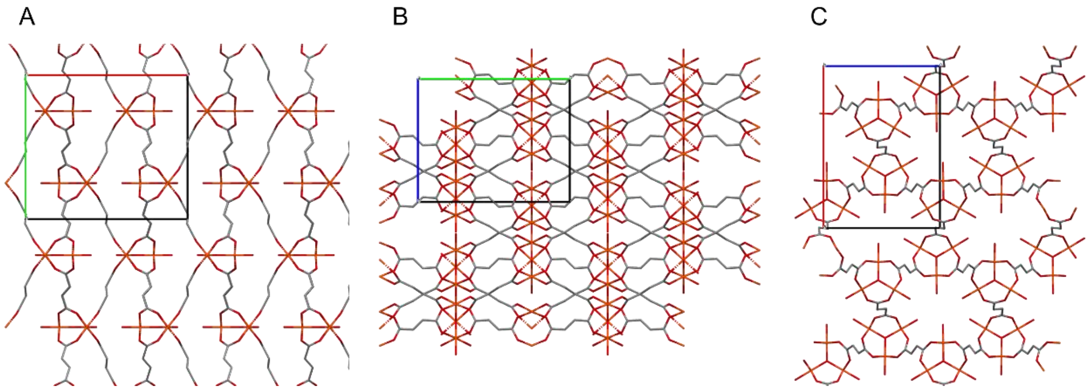

Figure S 25 Modeled structure of the γ phase of MIL-88 A.

Pore volume analysis of MIL-88 A phases

The micropore volume of each MIL-88 A phase is estimated by the MoloVol ver. 1.1.1 program[6] based on the corresponding crystal structural models. The structural models are scanned with a probe sphere with the radius of 1.4 Å, and the pore volume is evaluated as the volume occupied with this probe sphere. As summarized in the table below, the estimated pore volumes decreased in the order of α, β, and γ phases, in consistent to the observed phase transformation upon water desorption.

Table S 3 Simulated pore volumes for the different phases of MIL-88 A.

MIL-88 A Phase	Probe occupied volume (cm^3/g)	Unit cell fraction
**α**	0.594	0.601
**β**	0.576	0.591
**γ**	0.412	0.491

SEM/EDX

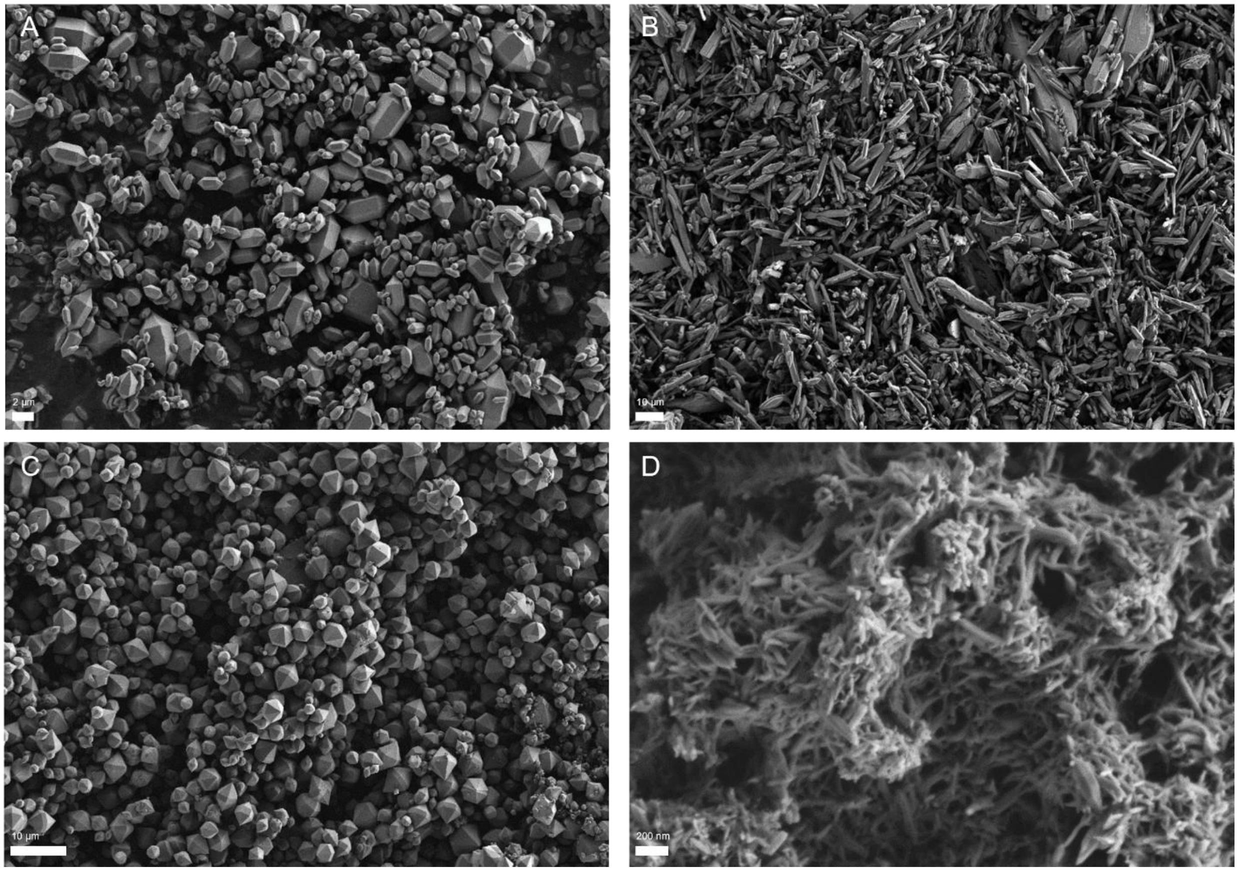

Figure S 26 SEM images of MIL 88 A synthesized via microwave (A; scale bar: 2 µm), hydrothermal (B; scale bar: 10 µm), solvothermal (C; scale bar: 10 µm), and mechanic (D; scale bar: 200 nm) synthesis approach.

We analyzed the crystal size distribution and morphologies by SEM. The microwave reaction gave spindle-like hexagonal MIL-88 A with a length of 1.99 µm ± 0.93 µm and a width of 1.01 µm ± 0.51 µm. Mostly hexagonal bipyramidal shaped crystals, also known as diamond-like in the literature,[7] (Length: 3.87 µm ± 1.53 µm; width: 3.37 µm ± 1.26 µm) were obtained by the solvothermal reaction. By hydrothermal synthesis mostly rod-like crystals were obtained with a length of 4.20 µm ± 3.66 µm and a width of 1.34 µm ±0.65 µm. MIL-88 A Mec shows a very broad crystal size distribution with nanocrystals with less than 200 nm in length and a few crystals with over 10 µm in length. This indicates that the size and shape of MIL-88 A can be easily tuned by the chosen reaction conditions but also provides a parameter that might impact the response towards exposure to water.

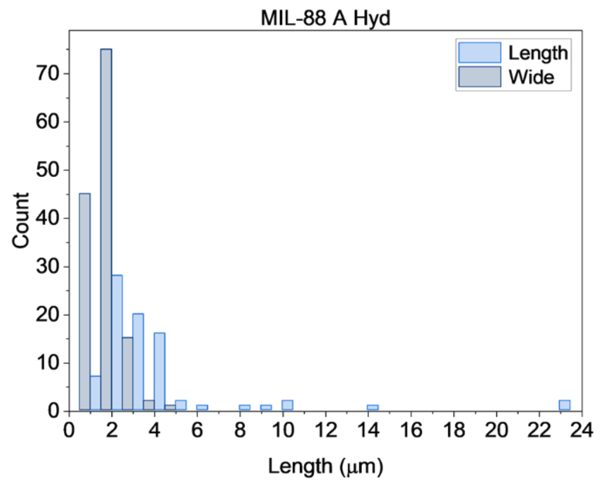

Figure S 27 Size distribution of MIL-88 A Hyd particles.

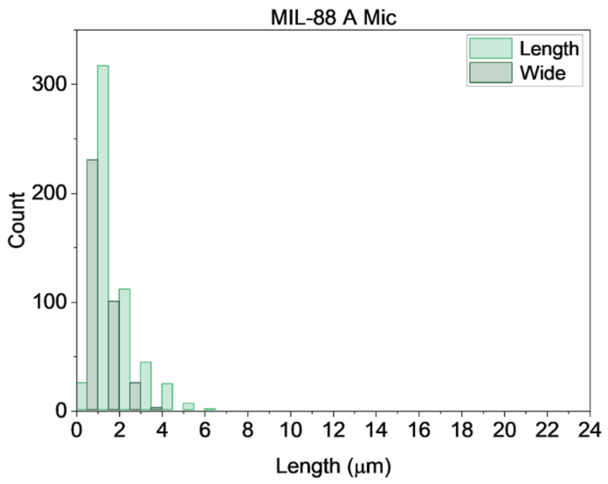

Figure S 28 Size distribution of MIL-88 A Mic particles.

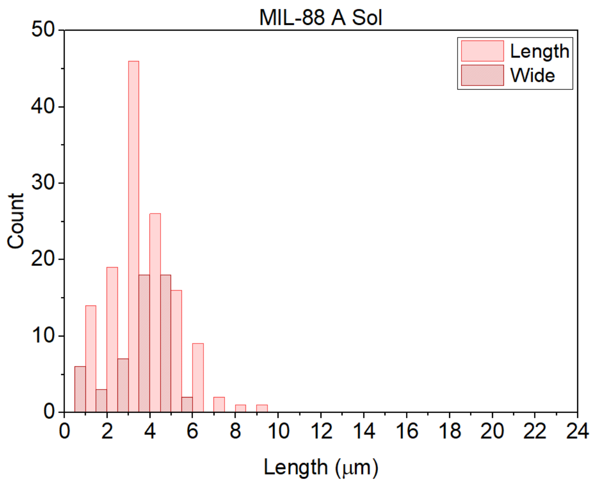

Figure S 29 Size distribution of MIL-88 A Sol particles.

Raman

Table S 4 Summarized peak position in Raman spectra (in cm^{-1}) of all dry and water samples and their assigned vibrational modes.[8]

Sample Code								
MIL-88 A Mec-dry	MIL-88 A Mec-H_2O	MIL-88 A Mic-dry	MIL-88 A Mic-H_2O	MIL-88 A Hyd-dry	MIL-88 A Hyd-H_2O	MIL-88 A Sol-dry	MIL-88 A Sol-H_2O	Vib. mode
1650.1 (vs)	1656.6 (vs)	1647.6 (vs)	1651.5 (vs)	1644.5 (vs)	1652.4 (vs)	1645.3 (vs)	1651.8 (vs)	ν_s(C=C)
	1588.4 (vw)		1588.4 (vw)		1587.3 (vw)		1587.9 (vw)	ν_{as}(C=O)
1524.8 (m)	1526.7 (m)	1530.2 (m)	1524.5 (m)	1526.2 (m)	1526.7 (m)	1528.5 (m)	1525.6 (m)	ν_{as}(C=O)
1430.9 (s)*	1432.5 (s)*	1430.8 (s)*	1430.7 (s)*	1429.9 (s)*	1431.5 (s)*	1430.6 (s)*	1431.5 (s)*	ν_s(C=O)
1274 (s)	1279.2 (s)	1271.4 (s)	1276.1 (s)	1272.5 (s)	1276.1 (s)	1271.1 (s)	1276.4 (s)	δ(C-O) in plane
1000.2 (vw)	1001.7 (w)	1000.2 (vw)	1000.8 (w)	998.2 (vw)	1000.4 (w)	998.6 (vw)	1002.1 (w)	δ(=C-H) out of plane
903.1 (s)	907.8 (s)	902.9 (s)	906.9 (s)	903.5 (s)	907.4 (s)	903.3 (s)	907.3 (s)	ω(=C-H) wagging
762.2 (s)	770.0 (s)	767.5 (s)	769.5 (s)	764.2 (s)	769.6 (s)	764.2 (s)	769.5 (s)	ρ(C-O) rocking
670.4 (w)	607.1 (w)	669.1 (w)	607.9 (w)	669.7 (w)	607.1 (w)	666.4 (w)	607.9 (w)	ω(-CH, -OH, O-C=O) wagging
499.6 (m)*	526.5 (m)	497.1 (m)*	528.8 (m)	495.6 (m)*	528.2 (m)	497.6 (m)*	527.6 (m)	mix of different vibrational modes including Fe-O cluster vibrations and lattice vibrational modes
422.8 (m)	439.1 (m)*	423.1 (m)	441.4 (m)*	421.3 (m)	443.1 (m)*	419.9 (m)	444.7 (m)*	
	287.1 (w)		290.1 (w)		290.1 (w)		286.6 (w)	
235.3 (m)	216.8 (m)	238.6 (s)	217.5 (m)	240.3 (s)	216.7 (m)	238.9 (s)	216.7 (m)	
	157.9 (sh)		167.2 (sh)		168.5 (sh)		165.9 (sh)	
136.2 (vs)	125.2 (s)	136.2 (vs)	122.6 (s)	139.5 (vs)	123.9 (s)	137.9 (vs)	126.5 (s)	

vs = very strong, s = strong, m = medium, w = weak, vw = very weak, and sh = shoulder
ν_s - symmetric stretch, ν_{as} - asymmetric stretch, δ - bending vibration
*multiple peaks present

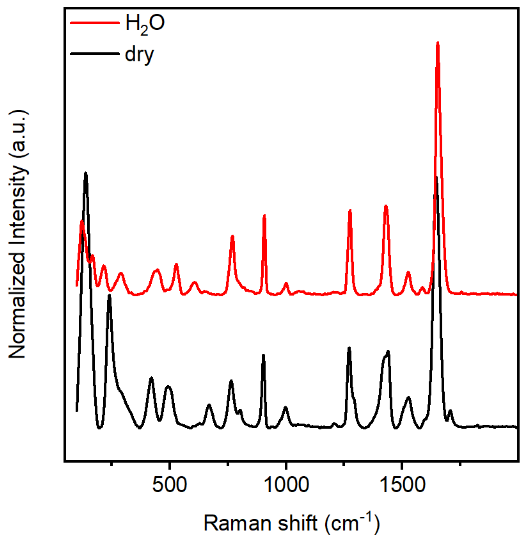

Figure S 30 Raman spectra of the dried (black) and solvated (red) MIL-88 A Hyd.

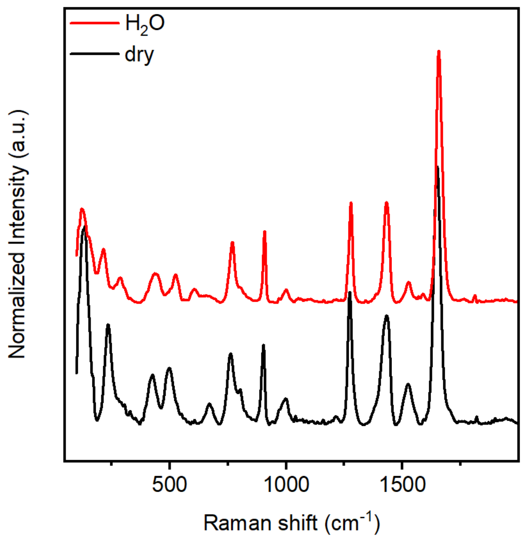

Figure S 31 Raman spectra of the dried (black) and solvated (red) MIL-88 A Mec.

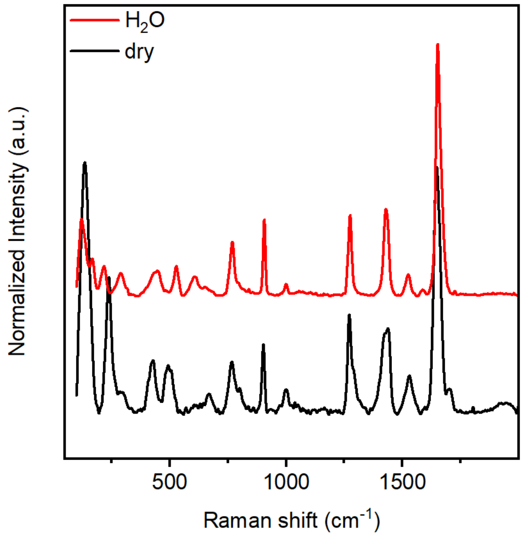

Figure S 32 Raman spectra of the dried (black) and solvated (red) MIL-88 A Mic.

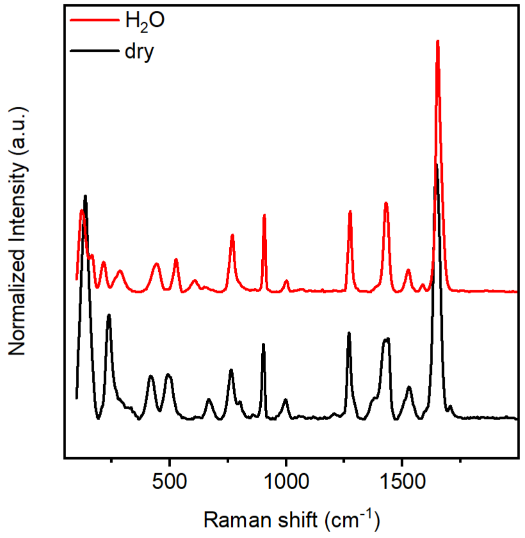

Figure S 33 Raman spectra of the dried (black) and solvated (red) MIL-88 A Sol.

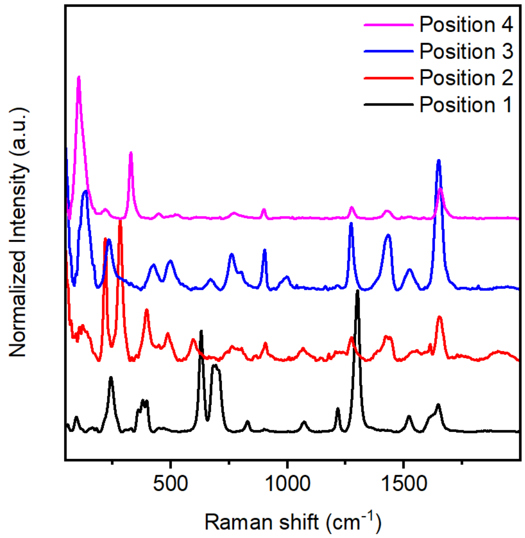

Figure S 34 Raman spectra of MIL-88 A Mec at different positions.

Figure S 34 presents the Raman spectra of a single MIL 88 A Mec sample were examined. It should be noted that several samples were analyzed during the course of this study. The presence of the impurities was detected in this particular sample. The behavior exhibited by this sample in the other analytical techniques did not differ from that exhibited by the other samples. This discrepancy can be explained by the fact that Raman spectroscopy is a surface-sensitive method

References

[1] T. Chalati, P. Horcajada, R. Gref, P. Couvreur, C. Serre, *Journal of Materials Chemistry* **2011**, *21*, 2220-2227.

[2] H. Jeong, J. Lee, *European Journal of Inorganic Chemistry* **2019**, *2019*, 4597-4600.

[3] O. Dolomanov, L. Bourhis, R. Gildea, J. Howard, H. Puschmann, *Journal of Applied Crystallography* **2009**, *42*, 339-341.

[4] G. M. Sheldrick, *Acta Crystallographica Section C: Structural Chemistry* **2015**, *71*, 3-8.

[5] P. Iacomi, B. Zheng, S. Krause, S. Kaskel, G. Maurin, P. L. Llewellyn, *Chemistry of Materials* **2020**, *32*, 3489-3498.

[6] J. B. Maglic, R. Lavendomme, *Journal of Applied Crystallography* **2022**, *55*, 1033-1044.

[7] L. Wang, Y. Zhang, X. Li, Y. Xie, J. He, J. Yu, Y. Song, *Scientific Reports* **2015**, *5*, 14341.

[8] a) J. Tittel, F. Knechtel, E. Ploetz, *Advanced Functional Materials* **2024**, *34*, 2307518; b) A. Benítez, J. Amaro-Gahete, D. Esquivel, F. Romero-Salguero, J. Morales, Á. Caballero, *Nanomaterials* **2020**, *10*, 424.
